# Review of Marine Cyanobacteria and the Aspects Related to Their Roles: Chemical, Biological Properties, Nitrogen Fixation and Climate Change

**DOI:** 10.3390/md21080439

**Published:** 2023-08-03

**Authors:** Hesham R. El-Seedi, Mohamed F. El-Mallah, Nermeen Yosri, Muaaz Alajlani, Chao Zhao, Muhammad A. Mehmood, Ming Du, Hammad Ullah, Maria Daglia, Zhiming Guo, Shaden A. M. Khalifa, Qiyang Shou

**Affiliations:** 1 International Research Center for Food Nutrition and Safety, Jiangsu University, Zhenjiang 212013, China; 2 International Joint Research Laboratory of Intelligent Agriculture and Agri-Products Processing, Jiangsu University, Jiangsu Education Department, Nanjing 210024, China; 3 Department of Chemistry, Faculty of Science, Islamic University of Madinah, Madinah 42351, Saudi Arabia; 4 Department of Chemistry, Faculty of Science, Menoufia University, Shebin El-Kom 32512, Egypt; elmallahmohammed9994@yahoo.com; 5 Chemistry Department of Medicinal and Aromatic Plants, Research Institute of Medicinal and Aromatic Plants (RIMAP), Beni-Suef University, Beni-Suef 62514, Egypt; nermeen.yosri@rimp.bsu.edu.eg; 6 China Light Industry Key Laboratory of Food Intelligent Detection & Processing, School of Food and Biological Engineering, Jiangsu University, Zhenjiang 212013, China; guozhiming@ujs.edu.cn; 7 Faculty of Pharmacy, Al-Sham Private University, Damascus 0100, Syria; muaaz.alajlani.foph@aspu.edu.sy; 8 College of Marine Sciences, Fujian Agriculture and Forestry University, Fuzhou 350002, China; zhchao@live.cn; 9 Bioenergy Research Center, Department of Bioinformatics and Biotechnology, Government College University Faisalabad, Faisalabad 38000, Pakistan; 10 School of Food Science and Technology, National Engineering Research Center of Seafood, Dalian Polytechnic University, Dalian 116034, China; duming@dlpu.edu.cn; 11 Department of Pharmacy, University of Napoli Federico II, Via D. Montesano 49, 80131 Naples, Italy; 12 Psychiatry and Psychology Department, Capio Saint Göran’s Hospital, Sankt Göransplan 1, 112 19 Stockholm, Sweden; 13 Second Clinical Medical College, Jinhua Academy, Zhejiang Chinese Medical University, Hangzhou 310053, China

**Keywords:** marine cyanobacteria, historical record, climate change, nitrogen fixation, secondary metabolites, clinical trials

## Abstract

Marine cyanobacteria are an ancient group of photosynthetic microbes dating back to 3.5 million years ago. They are prolific producers of bioactive secondary metabolites. Over millions of years, natural selection has optimized their metabolites to possess activities impacting various biological targets. This paper discusses the historical and existential records of cyanobacteria, and their role in understanding the evolution of marine cyanobacteria through the ages. Recent advancements have focused on isolating and screening bioactive compounds and their respective medicinal properties, and we also discuss chemical property space and clinical trials, where compounds with potential pharmacological effects, such as cytotoxicity, anticancer, and antiparasitic properties, are highlighted. The data have shown that about 43% of the compounds investigated have cytotoxic effects, and around 8% have anti-trypanosome activity. We discussed the role of different marine cyanobacteria groups in fixing nitrogen percentages on Earth and their outcomes in fish productivity by entering food webs and enhancing productivity in different agricultural and ecological fields. The role of marine cyanobacteria in the carbon cycle and their outcomes in improving the efficiency of photosynthetic CO_2_ fixation in the chloroplasts of crop plants, thus enhancing the crop plant’s yield, was highlighted. Ultimately, climate changes have a significant impact on marine cyanobacteria where the temperature rises, and CO_2_ improves the cyanobacterial nitrogen fixation.

## 1. Introduction

Cyanobacteria may be described as the pioneers of the planet Earth and are a one-of-a-kind gift of nature. The historical record dates the existence of cyanobacteria back to over 3.5 million years ago (MYA). They possess a number of natural qualities that make them suitable for several biotechnological applications [1,2,3]. Cyanobacteria (blue-green algae) are Gram-negative eubacteria. They are widely distributed throughout terrestrial, fresh water, wastewater, and marine habitats due to their immense morphological and physiological diversity [2,4]. Cyanobacteria come in a wide variety of morph types, such as species that are unicellular, surface-attached, as well as filamentous colony- and mat-forming. In the last few decades, the advent of ultrastructural and molecular methods has been evident as per the progress in taxonomic classification, the dominant tool for evaluating cyanobacterial diversity. These methods explain many yet unclear structures, adaptations, and phylogenetic relations within different clusters. The molecular methods rely on 16S rRNA gene sequencing as a standard, notably at the genus level. The 16S rRNA gene sequencing clusters closely correlate with traditional cyanobacterial taxa, which can be identified by various phenotypic characteristics [5,6]. Several species form significant symbiotic relationships with other micro- and macro-eukaryotes [7,8]. As life evolved on Earth, cyanobacteria played a pivotal role as the first organisms to engage in oxygenic photosynthesis [9]. In addition to their evolutionary niche, cyanobacteria have also played vital roles in the biogeochemical cycle of carbon (C) and nitrogen (N). An estimated 20–30% of the organic carbon present on Earth is derived from photosynthetic carbon fixation by cyanobacteria. Cyanobacteria also represent the primary N_2_-fixing microorganisms in marine environments [10,11,12].

Natural products have historically been used to inspire and develop new medications, and this remains one of the most successful methods for the discovery of small molecules for drug development [13]. Cyanobacteria are a unique source of small molecules, since they are thought to be one of the oldest forms of life on Earth and may thus constitute chemical factories with highly evolved secondary metabolite synthesis machinery. They have also been demonstrated to influence the biosynthesis of chemicals in marine invertebrates, such as sponges, ascidians, and shell-less mollusks, via endosymbiosis or diet-derived enrichment [13,14,15,16]. To date, more than 2000 secondary metabolites have been identified from marine cyanobacteria, especially *Moorea*, *Lyngbya*, and *Okeania* spp. [17,18]. Marine cyanobacteria have attracted the attention of many scientists in the fields of medicinal chemistry and pharmacology [19], as they demonstrate powerful biological activities such as antibacterial [20], antifungal [21], anticancer [22], and cytotoxic activities [23], as well as immunosuppression [24], anti-inflammatory [25], and antioxidant properties [26]. For instance, apratoxin D is a potent bioactive compound that is isolated from the marine cyanobacterium *Lyngbya* sp. and has demonstrated significant cytotoxicity against human lung cancer cells [27]. Four bioactive compounds, Dudawalamides A-D, isolated from *Moorea producens* were proven to be effective antiparasitic agents. All four of these compounds demonstrated potent activity, with IC_50_ ≥ 10 mM [28].

In terms of biological properties, cyanobacteria are dominant agents in the Earth’s nitrogen cycle. They use nitrogenase enzymes to reduce N_2_ to NH_3_ [29]. Because the nitrogenase enzyme complex is O_2_-sensitive, cyanobacteria spatially separate CO_2_ fixation from N_2_ fixation. Some cyanobacteria have developed specialized cellular structures known as heterocyst, which contain the activity of the nitrogenase enzyme and prevent it from being inactivated by O_2_ interaction during photosynthesis [30]. Accordingly, *Trichodesmium*, one of the marine cyanobacteria, is considered the dominant N_2_-fixing organism in the ocean. It accounts for around 50% of global natural N_2_ fixation [31]. While various studies have explained the mechanism of *Trichodesmium* N_2_ fixation, the mechanism of N_2_ fixation for other marine cyanobacteria is still unexplained. As part of our ongoing projects on chemical and pharmacological studies of marine natural products [32,33,34,35], we hereby summarize the historical and existence records of marine cyanobacteria, based on fossil documentation through the different eras, and their historical evolutionary records. We also screened for potent secondary metabolites from marine cyanobacteria, reported as possessing significant pharmacological activities in the 2017–2022 period, making note of any ongoing clinical trials during the said period. Finally, we highlighted the role of different groups of marine cyanobacteria in both nitrogen fixation and their outcomes, along with carbon fixation and in correlation to climate changes.

## 2. History and Existence

**Figure 1:**
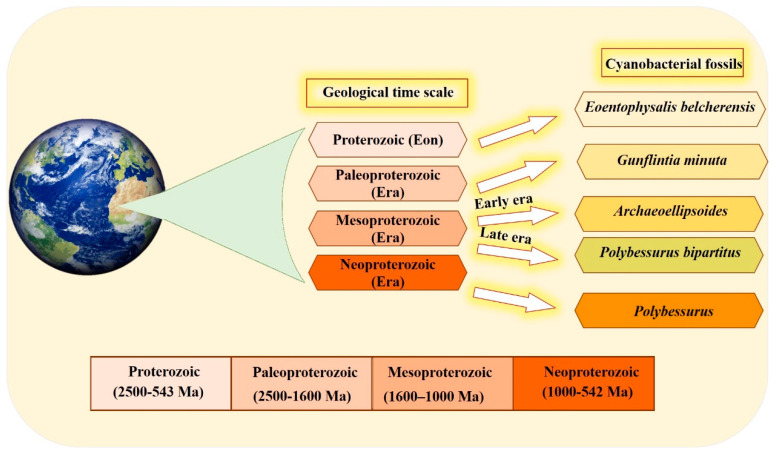
Timeline of the different cyanobacterial fossils recorded through different eras, mainly over the period ranging from 2500 to 542 million years ago, using a geological time scale.

The organized structure and differentiation in the morphology of cyanobacteria, and their dominance in paleontological history [36], are due to the vital role that the unique cyanobacterial metabolism played in ancient ecosystems. This is also influenced by the selectivity of preservation and fossilization for particular environments or cyanobacterial taxa. The cyanobacterial fossil record is one of the oldest, reaching back to approximately 3500 Ma [37]. Fossil identification occurs by comparing old samples (established specimens existing in the herbarium) with modern forms in the natural population [37,38]. Scientists have devoted their efforts to documenting cyanobacteria across different eras (Figure 1). Several studies have documented that the most ancient in disputable fossil cyanobacteria were recorded in the Proterozoic Eon. *Eoentophysalis belcherensis* Hofmann was the first colonial coccoid microfossil present in this period. Their existence has been reported in intertidal and shallow subtidal environments on a carbonate platform in Canada [38,39]. Other fossils were recorded by Lanier (1989) as dating back to the Paleoproterozoic era. These fossils include vertically and horizontally oriented filaments, viz. *Gunflintiaminuta,* suggesting photoautotrophic cyanobacteria [40]. During the early Mesoproterozoic era, the cyanobacterial population, including entophysalidacean, oscillatoriacean, and nostocalean cyanobacteria, were widely spread across all possible ecological niches, from supratidal flats to open shelf marine environments [41,42]. The formation of distinct carbonate precipitates in this era helped fossilize a member of what was named the *Archaeoellipsoides* cyanobacterial genus in India [43]. Although they share many similarities with the extant *Synechococcus* genus, the samples show residues of akinetes resembling cyanobacteria *Nostoc* or *Stigonema* [36,44]. The late Mesoproterozoic era shows evidence of the existence of mat-dwelling and planktic chroococcacean cyanobacteria, with some cyanobacteria recognizable today dating back to the early Mesoproterozoic era. A distinctive feature of the evolution of cyanobacteria is the presence of a stalked cyanobacterium *Polybessurus bipartitus* [41]. The diversification of protists represents a critical event for developing the biosphere, and this has been documented as occurring in the era now named the Neoproterozoic era. Some new morphological varieties of cyanobacterial fossil debuted in this era, i.e., the remains of the stalked cyanobacterium *Polybessurus* Fairchild ex Green et al., which can sometimes be found in sediments [36]. In the near past, morphological criteria were solely used to classify cyanobacteria taxonomically; however, in the modern taxonomy, more adequate and complementary tools have been employed [45]. In the last few decades, the criteria of cyanobacterial taxonomic classification have radically changed after applying data obtained from electron microscopic studies along with phylogenetic analyses, mainly derived from molecular sequencing [46]. Modern techniques, such as the molecular method combined with traditional cyanobacterial morphological and ecological diversity studies, as well as the re-evaluation and redefinition of contemporary typical characters and markers of cyanobacterial entities in various biotopes, are inevitable taxonomic criteria for the primary classification. The molecular method used 16S rRNA gene sequencing as a standard, particularly at the genus level. The 16S rRNA gene sequencing clusters correspond to the traditional cyanobacterial taxa, which are identified by distinctive phenotypic features [5,6]. The combination of molecular methods with cyanobacterial taxonomy advanced important key aspects of cyanobacterial systematics as follows: Firstly, regarding various taxonomic units, the morphological characters should concur with their phylogenetic position. Secondly, phylogenetic relationships among cyanobacteria were discovered using combining molecular and phenotypic analyses, and a highly genetic variety was identified, particularly in coccoid and simple filamentous and pseudo-filamentous types [47]. Finally, a number of studies concluded that the fossil record of cyanobacteria, along with the modern methods of re-evaluation and redefinition of contemporary typical characters, helped scientists understand the complexity and diversity present in the history of evolution.

## 3. Bioactive Compounds Isolated from Marine Cyanobacteria

**Figure 2:**
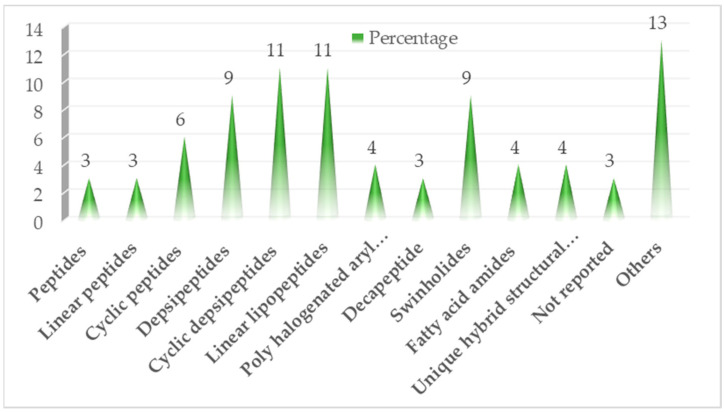
Flowchart showing classes of screened bioactive compounds and their derivatives.

**Figure 3:**
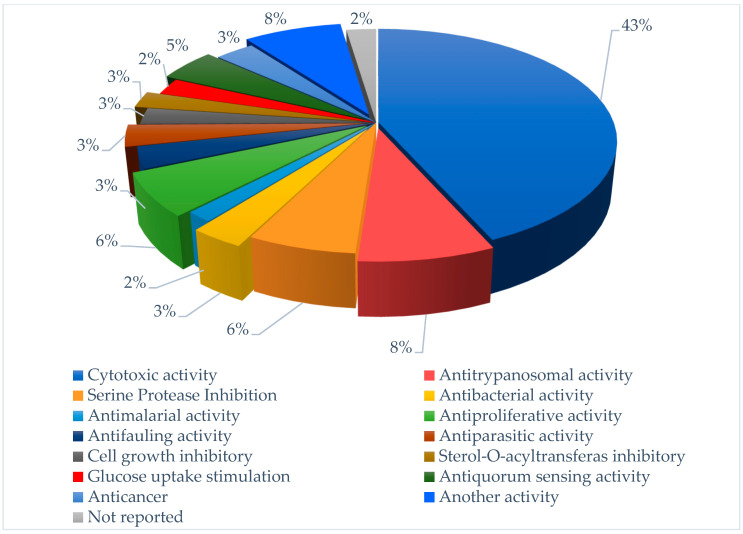
Flowchart reporting percentage of each pharmacological target for screened compounds in the last six years.

Marine cyanobacteria have valuable potent secondary metabolites, possessing significant pharmacological activities [13]. Here, we survey the different recently isolated compounds with various biological targets, i.e., cytotoxic activity, antiparasitic activity, serine protease inhibition, anticancer and cell proliferation activity, anti-quorum sensing activity, and antibacterial activity of the period between 2017 and 2022 as illustrated in Table 1, Table 2, Table 3, Table 4, Table 5, Table 6 and Table 7 and Figure 2 and Figure 3. It is noteworthy to mention that we described in more detail the highly bioactive compounds. 

Of approximately 83 compounds screened in the last six years, most of them are of peptide origin (Figure 2), and 43% of them showed significant cytotoxicity, with about 8% showing potency in anti-trypanosomal activity as shown in Figure 3.

### 3.1. Cytotoxic Activity

**Scheme 1:**
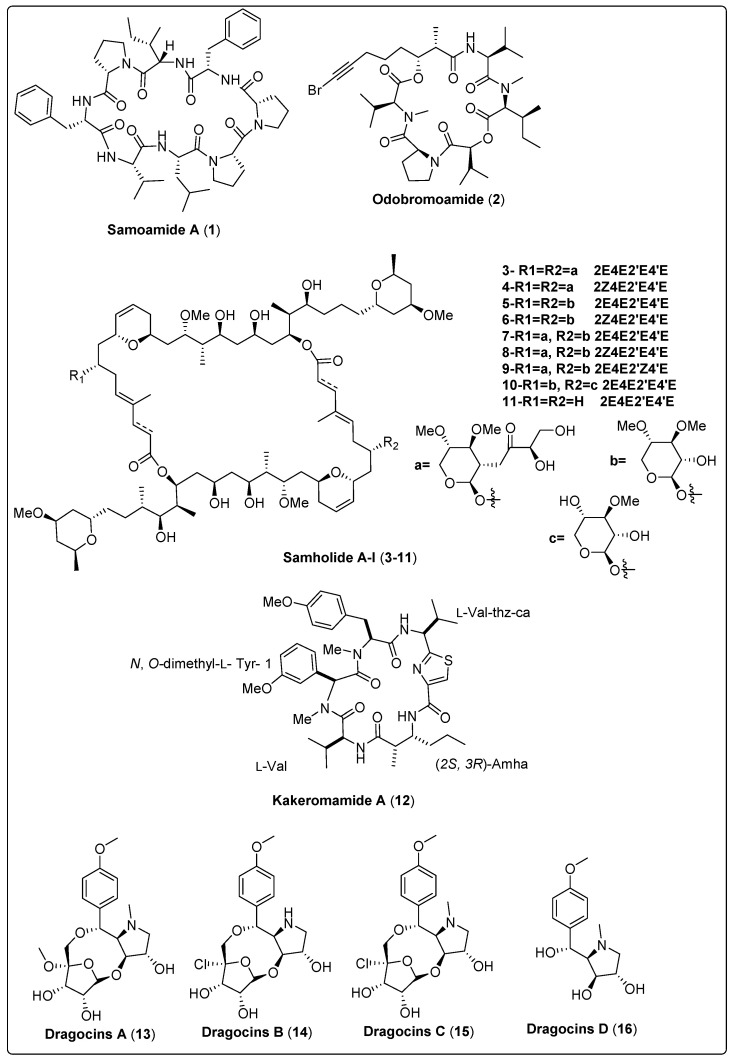

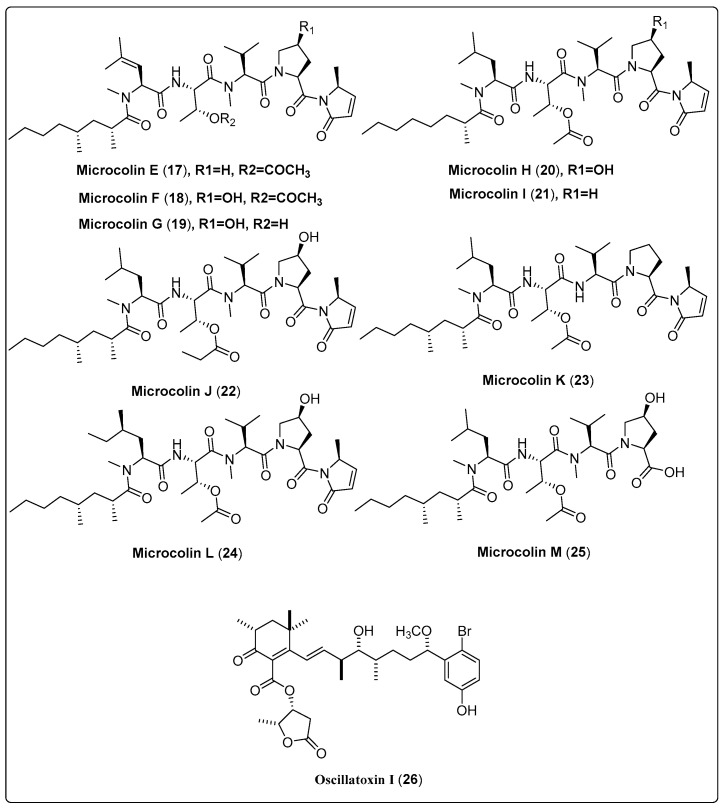
Structures of isolated cyanobacterial compounds from 2017 to 2022 with cytotoxicity targets.

Samoamide A (**1**), a natural cyclic octapeptide, was isolated from marine cyanobacteria cf. *Symploca* sp. It showed a significant cytotoxic effect against H460 and H116 cells, as confirmed by the MTT assay. Compound (**1**) exhibited IC_50_ values of 1.1 µM and 4.5 µM for H460 and H116 cells, respectively, in comparison with the positive control (doxorubicin with IC_50_ value of 0.2 µM). The mechanism of action was explained by the inhibition of the enzyme dipeptidyl peptidase (CD26, DPP4) at a reported allosteric binding site [48]. In another study, the marine cyanobacteria *Okeania* sp. yielded a bioactive compound named Odobromoamide (**2**). This study assessed cytotoxic activity against human lung carcinoma (H-460) in-vitro using an MTT assay. Bioactive compound (**2**) shows a significant effect on HeLa S3 cells with IC_50_ value 0.31 μM. The cytotoxicity associated with this compound was dependent on the caspase family of proteins [49]. Nine new swinholide-related compounds, named Samholide A–I (**3**–**11**), were isolated from cf. *Phormidium* sp. The cytotoxic activity of these new compounds was tested against a human lung cancer cell line (H-460). All nine compounds demonstrated obvious activity, with IC_50_ lower than 1 µM. Compounds (**3**, **6**, **7**, and **8**) show potent IC_50s_ of 0.17 ± 0.01, 0.17 ± 0.06, 0.17 ± 0.01, and 0.17 ± 0.00 µM in comparison with the positive control (doxorubicin IC_50_ of 0.30 ± 0.02 µM) [50]. Nakamura and his colleagues (2018) isolated the bioactive compound Kakeromamide A (**18**) from the marine cyanobacteria *Moorea bouillonii*. This study assessed the molecular mechanism of (**18**) on cytotoxic activity using HeLa cell lines. The findings reported that compound (**18**) had a significant effect with IC_50_ of 10 μM. The mechanism of action of kakeromamide A displays the differentiation of neural stem cells into astrocytes [51]. Nine bioactive compounds, Microcolin E–M (**17**–**25**), were isolated from the marine cyanobacteria *Moorea producens*. Cytotoxic activity was tested in-vitro using human lung carcinoma (H460). The results reported that compounds (**18**, **20**, and **22**) have potent activity with IC_50_ ranged between 37 ± 4 nM and 69 ± 10 nM against human lung carcinoma (H460). Significant cancer cell cytotoxic activity supports the investigation of the mechanism of cytotoxic action [52]. We conclude that the most potent bioactive compounds with potent cytotoxic activity as shown in Table 1, Scheme 1 and their spectral analysis reviewed in Table S1 are in need of more in-depth investigation. In-vivo studies are also highly recommended for obtaining promising lead compounds as anticancer drugs.

**Table 1:** Screening for bioactive compound cytotoxicity targets isolated from marine cyanobacteria between 2017 and 2022.

Compound Name/Class	Source/Place and DATE	Separation Tools	Pharmacological Activity	Reference
Samoamide A, cyclic octapeptide (**1**)	cf. *Symploca* sp./Vatia Bay, American Samoa (July 2014)	HPLC, SPE and VLC	Cytotoxic activity Model: H460 cells and H116 cells. Assay: MTT assay IC_50_ = 1.1 mM for H460 cells IC_50_ = 4.5 mM for H116 cells PC: Doxorubicin (IC_50_ = 0.2 µM NC: DMSO	[48]
Odobromoamide, cyclo-depsipeptide (**2**)	*Okeania* sp./Odo, Okinawa Prefecture, Japan (May 2009)	HPLC and ODS-CC	Cytotoxic activity Model: HeLa S3 cells Assay: MTT assay IC_50_ = 0.31 µM	[49]
Samholide A, swinholide (**3**)	*Phormidium* sp./Fagaalu Park in American Samoa, U.S. (July 2014)	VLC, SPE, RP-HPLC and HPLC	Cytotoxic activity Model: Human lung carcinoma (H-460) Assay: MTT assay IC_50_ = 0.17 ± 0.01 µM PC: doxorubicin (IC_50_ = 0.30 ± 0.02 µM)	[50]
Samholide B, swinholide (**4**)	*Phormidium* sp./Fagaalu Park in American Samoa, U.S. (July 2014)	VLC, SPE, RP-HPLC and HPLC	Cytotoxic activity Model: Human lung carcinoma (H-460) Assay: MTT assay IC_50_ = 0.52 ± 0.02 µM PC: doxorubicin (IC_50_ = 0.30 ± 0.02 µM)	[50]
Samholide C, swinholide (**5**)	*Phormidium* sp./Fagaalu Park in American Samoa, U.S. (July 2014)	VLC, SPE, RP-HPLC and HPLC	Cytotoxic activity Model: Human lung carcinoma (H-460) Assay: MTT assay IC_50_ = 0.21 ± 0.08 µM PC: doxorubicin (IC_50_ = 0.30 ± 0.02 µM)	[50]
Samholide D, swinholide (**6**)	*Phormidium* sp./Fagaalu Park in American Samoa, U.S. (July 2014)	VLC, SPE, RP-HPLC and HPLC	Cytotoxic activity Model: Human lung carcinoma (H-460) Assay: MTT assay IC_50_ = 0.17 ± 0.06 µM PC: doxorubicin (IC_50_ = 0.30 ± 0.02 µM)	[50]
Samholide E, swinholide (**7**)	*Phormidium* sp./Fagaalu Park in American Samoa, U.S. (July 2014)	VLC, SPE, RP-HPLC and HPLC	Cytotoxic activity Model: Human lung carcinoma (H-460) Assay: MTT assay IC_50_ = 0.17 ± 0.01 µM PC: doxorubicin (IC_50_ = 0.30 ± 0.02 µM)	[50]
Samholide F, swinholide (**8**)	*Phormidium* sp./Fagaalu Park in American Samoa, U.S. (July 2014)	VLC, SPE, RP-HPLC and HPLC	Cytotoxic activity Model: Human lung carcinoma (H-460) Assay: MTT assay IC_50_ = 0.17 ± 0.00 µM PC: doxorubicin (IC_50_ = 0.30 ± 0.02 µM)	[50]
Samholide G, swinholide (**9**)	*Phormidium* sp./Fagaalu Park in American Samoa, U.S. (July 2014)	VLC, SPE, RP-HPLC and HPLC	Cytotoxic activity Model: Human lung carcinoma (H-460) Assay: MTT assay IC_50_ = 0.21 ± 0.01 µM PC: doxorubicin (IC_50_ = 0.30 ± 0.02 µM)	[50]
Samholide H, swinholide (**10**)	*Phormidium* sp./Fagaalu Park in American Samoa, U.S. (July 2014)	VLC, SPE, RP-HPLC and HPLC	Cytotoxic activity Model: Human lung carcinoma (H-460) Assay: MTT assay IC_50_ = 0.47 ± 0.04 µM PC: doxorubicin (IC_50_ = 0.30 ± 0.02 µM)	[50]
Samholide I, swinholide (**11**)	*Phormidium* sp./Fagaalu Park in American Samoa, U.S. (July 2014)	VLC, SPE, RP-HPLC and HPLC	Cytotoxic activity Model: Human lung carcinoma (H-460) Assay: MTT assay IC_50_ = 0.91 ± 0.05 µM PC: doxorubicin (IC_50_ = 0.30 ± 0.02 µM)	[50]
Kakeromamide A, cyclic pentapeptide (**12**)	*Moorea bouillonii*/Kakeroma Island in Kagoshima, Japan	RP-HPLC	Cytotoxic activity Model: HeLa cells IC_50_ = 10 µM PC: Not reported	[51]
Dragocin A, uniquehybrid structural class (**13**)	*Symploca*-like morphology/Boca del Drago, Panama	VLC and RP-HPLC	Cytotoxic activity Model: Lung cancer cells (H460) Survival: At 3 µg/mL concentration = 23% At 30 µg/mL concentration = 10% PC: doxorubicin (EC_50_ = 0.0236–0.0078 µM)	[53]
Dragocin B, uniquehybrid structural class (**14**)	*Symploca*-like morphology/Boca del Drago, Panama	VLC and RP-HPLC	Cytotoxic activity Model: Lung cancer cells (H460) Survival: At 3 µg/mL concentration > 140% At 30 µg/mL concentration ≃ 60% PC: doxorubicin (EC50 = 0.0236–0.0078 µM)	[53]
Dragocin C, uniquehybrid structural class (**15**)	*Symploca*-like morphology/Boca del Drago, Panama	VLC and RP-HPLC	Cytotoxic activity Model: Lung cancer cells (H460) Survival: At 3 µg/mL concentration ≃ 136% At 30 µg/mL concentration ≃ 100% PC: doxorubicin (EC50 = 0.0236–0.0078 µM)	[53]
Dragocin D, uniquehybrid structural class (**16**)	*Symploca*-like morphology/Boca del Drago, Panama	VLC and RP-HPLC	Cytotoxic activity Model: Lung cancer cells (H460) Survival: At 3 µg/mL concentration ≃ 77% At 30 µg/mL concentration ≃ 15% PC: doxorubicin (EC50 = 0.0236–0.0078 mM)	[53]
Microcolin E, linear lipopeptide (**17**)	*Moorea producens*/Playa Kalki, Curaçao (September 1997)	VLC and HPLC	Cytotoxic activity Model: lung carcinoma (H460) Assay: MTT IC_50_ = 1000 ± 20 nM PC: doxorubicin (IC_50_ = 180 ± 10 nM)	[52]
Microcolin F, linear lipopeptide (**18**)	*Moorea producens*/Playa Kalki, Curaçao (September 1997)	VLC and HPLC	Cytotoxic activity Model: lung carcinoma (H460) Assay: MTT IC_50_ = 37 ± 4 nM PC: doxorubicin (IC_50_ = 180 ± 10 nM)	[52]
Microcolin G, linear lipopeptide (**19**)	*Moorea producens*/Playa Kalki, Curaçao (September 1997)	VLC and HPLC	Cytotoxic activity Model: lung carcinoma (H460) Assay: MTT IC_50_ = 160 ± 20 nM PC: doxorubicin (IC_50_ = 180 ± 10 nM)	[52]
Microcolin H, linear lipopeptide (**20**)	*Moorea producens*/Playa Kalki, Curaçao (September 1997)	VLC and HPLC	Cytotoxic activity Model: lung carcinoma (H460) Assay: MTT IC_50_ = 47 ± 5 nM PC: doxorubicin (IC_50_ = 180 ± 10 nM)	[52]
Microcolin I, linear lipopeptide (**21**)	*Moorea producens*/Playa Kalki, Curaçao (September 1997)	VLC and HPLC	Cytotoxic activity Model: lung carcinoma (H460) Assay: MTT IC_50_ = 550 ± 50 nM PC: doxorubicin (IC_50_ = 180 ± 10 nM)	[52]
Microcolin J, linear lipopeptide (**22**)	*Moorea producens*/Playa Kalki, Curaçao (September 1997)	VLC and HPLC	Cytotoxic activity Model: lung carcinoma (H460) Assay: MTT IC_50_ = 69 ± 10 nM PC: doxorubicin (IC_50_ = 180 ± 10 nM)	[52]
Microcolin K, linear lipopeptide (**23**)	*Moorea producens*/Playa Kalki, Curaçao (September 1997)	VLC and HPLC	Cytotoxic activity Model: lung carcinoma (H460) Assay: MTT IC_50_ = 200 ± 50 nM PC: doxorubicin (IC_50_ = 180 ± 10 nM)	[52]
Microcolin L, linear lipopeptide (**24**)	*Moorea producens*/Playa Kalki, Curaçao (September 1997)	VLC and HPLC	Cytotoxic activity Model: lung carcinoma (H460) Assay: MTT IC_50_ = not tested PC: doxorubicin (IC_50_ = 180 ± 10 nM)	[52]
Microcolin M, linear lipopeptide (**25**)	*Moorea producens*/Playa Kalki, Curaçao (September 1997)	VLC and HPLC	Cytotoxic activity Model: lung carcinoma (H460) Assay: MTT IC_50_ = 510 ± 60 nM PC: doxorubicin (IC_50_ = 180 ± 10 nM)	[52]
Oscillatoxin I, aplysiatoxin (**26**)	*Moorea producens*/Kuba Beach, Nakagusuku, Okinawa, Japan (July 2010)	RP-HPLC	Cytotoxic activity Model: Mouse leukemia cells (L1210) IC_50_ = 4.6 µg/mL Assay: XTT Concentration: 10 µg/mL	[54]

### 3.2. Antiparasitic Activity

**Scheme 2:**
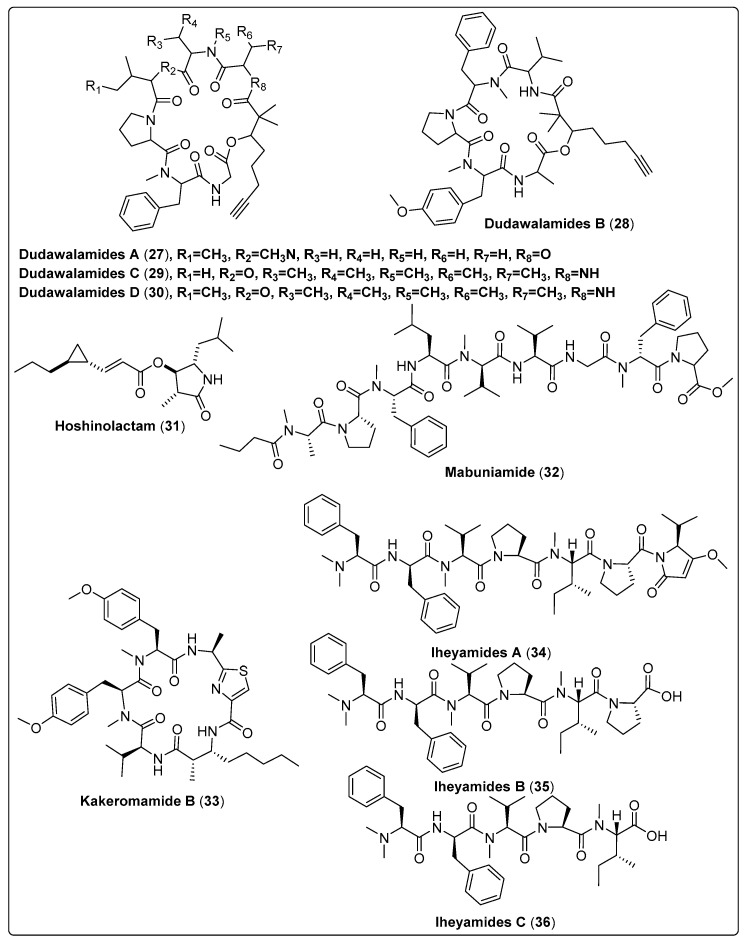

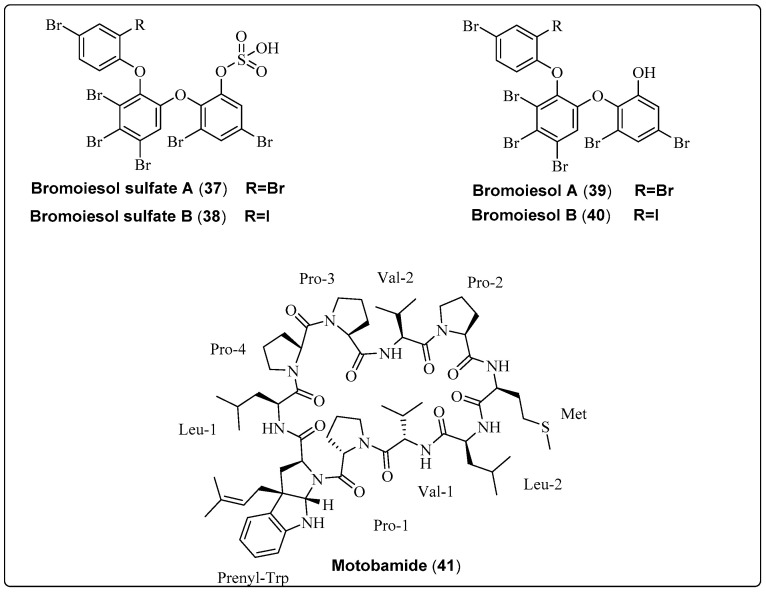
Structures of isolated cyanobacterial compounds from 2017 to 2022 with anti-trypansomal activity.

Four bioactive compounds, Dudawalamide A–D (**27**–**30**), were isolated from the marine cyanobacteria *Moorea producens*. The antiparasitic activity was tested in-vitro against malaria, Leishmaniasis, and Chagas disease. The results reported that compounds have potent activity against malaria, with IC_50_ ranged between 3.5 and 10 µM, and significant activity against Leishmaniasis, with IC_50_ > 10 µM. The mechanism of action for all compounds needs further investigation [28]. Ogawa and his colleagues isolated a bioactive compound named Hoshinolactam (**31**) from marine cyanobacteria. This compound showed a potent effect against *Trypanosoma brucei* with an IC_50_ value of 6.1 nM in comparison with the positive control (pentamidine with an IC_50_ value of 4.7 nM). We also assessed the cytotoxicity of human fetal lung fibroblast MRC-5 cells. The findings showed an acceptable IC_50_ value > 25,000 nM in comparison with the positive control (pentamidine with an IC_50_ value of 16,800 nM) [55]. Bioassay-guided fractionation in isolating the lipopeptide mabuniamide (**32**) from *Okeania* sp. Evaluation of antimalarial activity was conducted on a *Plasmodium falciparum* 3D7 clone in vitro. The results reported that compound (**32**) exhibits a good effect with an IC_50_ of 1.4 ± 0.2 µM when compared with the positive control (chloroquine; IC_50_ 7.6 ± 0.5 nM). This study did not report the mode of action for the evaluated compound [56]. Kakeromamide B (**33**), a new cyclic peptide, was isolated from marine cyanobacteria *Moorea producens*. It showed a significant antimalarial effect against blood-stage *Plasmodium falciparum* and liver-stage *P. berghei*. Findings reported that (**33**) exhibited high EC_50_ values of 0.89 and 1.1 µM in comparison with the positive control (atovaquone with EC_50_ values of 0.0061 and <0.00028 µM) for the blood stage and liver stage, respectively. Its mode of action was suggested to be binding to several *Plasmodium* actin-like proteins and a sortilin protein, providing possible interference with the invasion of the parasite to host cells. In addition, cytotoxic activity was evaluated using HEK293T and HepG2 human cell lines as models. Compound (**33**) has potent cytotoxicity with the same IC_50_ > 2.3 µM for both human cell lines, when compared with the positive control atovaquone IC_50_ values > 2.0 µM for HEK293T. The compounds were not tested for HepG2 [57]. Kurisawa et al. (2020) isolated three new linear peptides from *Dapis* sp., named iheyamides A (**34**), B (**35**), and C (**36**). These new compounds were evaluated for cytotoxicity and anti-trypanosomal activity using normal human cells (WI-38) (*Trypanosoma brucei rhodesiense* and *Trypanosoma brucei brucei*) as models. Their findings showed that (**34**) has a potent anti-trypanosomal and cytotoxicity to the parasite with IC_50_ values of 1.5 and 18 µM, respectively, compared with pentamide as a positive control, with IC_50_ ranging between 0.001 and 0.005 µM, while (**35**) and (**36**) have low activity with IC_50_ > 20 µM. The mechanism of action of compound (**34**) was determined owing to its growth-inhibitory activities against *T. b. rhodesiense* and *T. b. brucei*. [58]. Finally, from the survey, shown in Table 2, Scheme 2 and their spectral analysis reviewed in Table S2 we conclude that a highly potent bioactive compound, i.e., (**34**), may be a promising lead compound for a new drug.

**Table 2:** Screening for bioactive compound antiparasitic targets isolated from marine cyanobacteria between 2017 and 2022.

Compound Name/Class	Source/Place and Date	Separation Tools	Pharmacological Activity	Reference
Dudawalamide A, cyclic depsipeptide (**27**)	*Moorea producens*/Dudawali Bay in Papua New Guinea (in April 2006)	SPE, VLC and RP-HPLC	Antiparasitic activity Models: Malaria (*Plasmodium falciparum*) IC_50_: 3.6 µM PC: chloroquine leishmaniasis (*Leishmania donovani*) IC_50_: >10 µM PC: amphotericin B Chagas disease (*Trypanosoma cruzi*) GI: 12% µg/mL PC: benznidazole Concentrations: 10, 2, 0.4, and 0.08 µg/mL	[28]
Dudawalamide B, cyclic depsipeptide (**28**)	*Moorea producens*/Dudawali Bay in Papua New Guinea (in April 2006)	SPE, VLC and RP-HPLC	Antiparasitic activity Models: Malaria (*Plasmodium falciparum*) IC_50_: 8.0 µM PC: chloroquine leishmaniasis (*Leishmania donovani*) IC_50_: >10 µM PC: amphotericin B Chagas disease (*Trypanosoma cruzi*) GI: 7% µg/mL PC: benznidazole Concentrations: 10, 2, 0.4, and 0.08 µg/mL	[28]
Dudawalamide C, cyclic depsipeptide (**29**)	*Moorea producens*/Dudawali Bay in Papua New Guinea (in April 2006)	SPE, VLC and RP-HPLC	Antiparasitic activity Models: Malaria (*Plasmodium falciparum*) IC_50_: 10 µM PC: chloroquine leishmaniasis (*Leishmania donovani*) IC_50_ = Not tested PC: amphotericin B Chagas disease (*Trypanosoma cruzi*) GI = Not tested PC: benznidazole Concentrations: 10, 2, 0.4, and 0.08 µg/mL	[28]
Dudawalamide D, cyclic depsipeptide (**30**)	*Moorea producens*/Dudawali Bay in Papua New Guinea (in April 2006)	SPE, VLC and RP-HPLC	Antiparasitic activity Models: Malaria (*Plasmodium falciparum*) IC_50_: 3.5 µM PC: chloroquine leishmaniasis (*Leishmania donovani*) IC_50_: 2.6 µM PC: amphotericin B Chagas disease (*Trypanosoma cruzi*) GI: 60 µg/mL PC: benznidazole Concentrations: 10, 2, 0.4, and 0.08 µg/mL	[28]
Hoshinolactam, contain cyclopropane ring and a γ-lactam ring system (**31**)	Not reported/Hoshino, Okinawa	RP-HPLC	Anti-trypanosomal activity Model: *Trypanosoma brucei* IC_50_: 6.1 nM PC: pentamidine (IC_50_: 4.7 nM)	[55]
Mabuniamide, lipopeptide (**32**)	*Okeania* sp./Odo, Okinawa, Japan (May 2018)	HPLC	Antimalarial activity Model: *P. falciparium* (3D7 clone) IC_50_: 1.4 ± 0.2 μM PC: chloroquine (IC_50_: 7.6 ± 0.5 nM)	[56]
Kakeromamide B, cyclic peptide (**33**)	*Moorea producens*/Tuvuca Island in Fiji (September 2007)	VLC, SPE and HPLC	Antimalarial activity Model: Asexual blood-stage *Plasmodium falciparum* (EC_50_ = 0.89 µM) PC: Atovaquone (EC_50_ = 0.0061 µM) Liver-stage *P. berghei* (EC_50_ = 1.1 µM) PC: Atovaquone (EC_50_: <0.00028 µM)	[57]
Iheyamide A, linear peptide (**34**)	*Dapis* sp./Noho Island, Iheya Village, Okinawa, Japan (August 2019)	RP-HPLC	Anti-trypansomal Model: *Trypanosoma brucei rhodesiense* IC_50_:1.5 μM PC: Pentamide (IC_50_: 0.005 μM) *Trypanosoma brucei brucei* IC_50_: 1.5 μM PC: Pentamide (IC_50_: 0.001 μM)	[58]
Iheyamide B, linear peptide (**35**)	*Dapis* sp./Noho Island, Iheya Village, Okinawa, Japan (August 2019)	RP-HPLC	Anti-trypansomal Model: *T. brucei rhodesiense* IC_50_: >20 μM PC: Pentamide (IC_50_: 0.005) *T. b. brucei* IC_50_: >20 μM PC: Pentamide (IC_50_: 0.001)	[58]
Iheyamide C, linear peptide (**36**)	*Dapis* sp./Noho Island, Iheya Village, Okinawa, Japan (August 2019)	RP-HPLC	Anti-trypansomal Model: *T. b. rhodesiense* IC_50_: >20 μM PC: Pentamide (IC_50_: 0.005) *T. b. brucei* IC_50_: >20 μM PC: Pentamide (IC_50_: 0.001)	[58]
Bromoiesol sulfate A, polyhalogenated aryl sulfate (**37**)	*Salileptolyngbya* sp./Ie-Island, Okinawa, Japan (September 2020)	HPLC	Anti-trypansomal activity Model: bloodstream *Trypanosoma brucei Rhodesiense* Assay: Anti-trypansomal assay IC_50_: 8.8 ± 1.3 μM PC: NR NC: NR	[59]
Bromoiesol sulfate B, polyhalogenated aryl sulfate (**38**)	*Salileptolyngbya* sp./Ie-Island, Okinawa, Japan (September 2020)	HPLC	Anti-trypansomal activity Model: bloodstream *Trypanosoma brucei Rhodesiense* Assay: Anti-trypansomal assay IC_50_: 7.9 ± 1.8 μM PC: NR NC: NR	[59]
Bromoiesol A, polyhalogenated aryl (**39**)	*Salileptolyngbya* sp./Ie-Island, Okinawa, Japan (September 2020)	HPLC	Anti-trypansomal activity Model: bloodstream *Trypanosoma brucei Rhodesiense* Assay: Anti-trypansomal assay IC_50_: 1.2 ± 0.1 μM PC: NR NC: NR	[59]
Bromoiesol B, polyhalogenated aryl (**40**)	*Salileptolyngbya* sp./Ie-Island, Okinawa, Japan (July 2020)	HPLC	Anti-trypansomal activity Model: bloodstream *Trypanosoma brucei Rhodesiense* Assay: Anti-trypansomal assay IC_50_: 0.70 ± 0.23 μM PC: NR NC: NR	[59]
Motobamide, cyclic peptide (**41**)	*Leptolyngbya* sp./Bise, Okinawa Island, Okinawa Prefecture, Japan (April 2018)	HPLC	Anti-trypansomal activity *Trypanosoma brucei rhodesiense* strains IL-1501 Assay: Anti-trypansomal Model: bloodstream IC_50_: 2.3 μM	[60]

### 3.3. Serine Proteases Inhibitory Effect

**Scheme 3:**
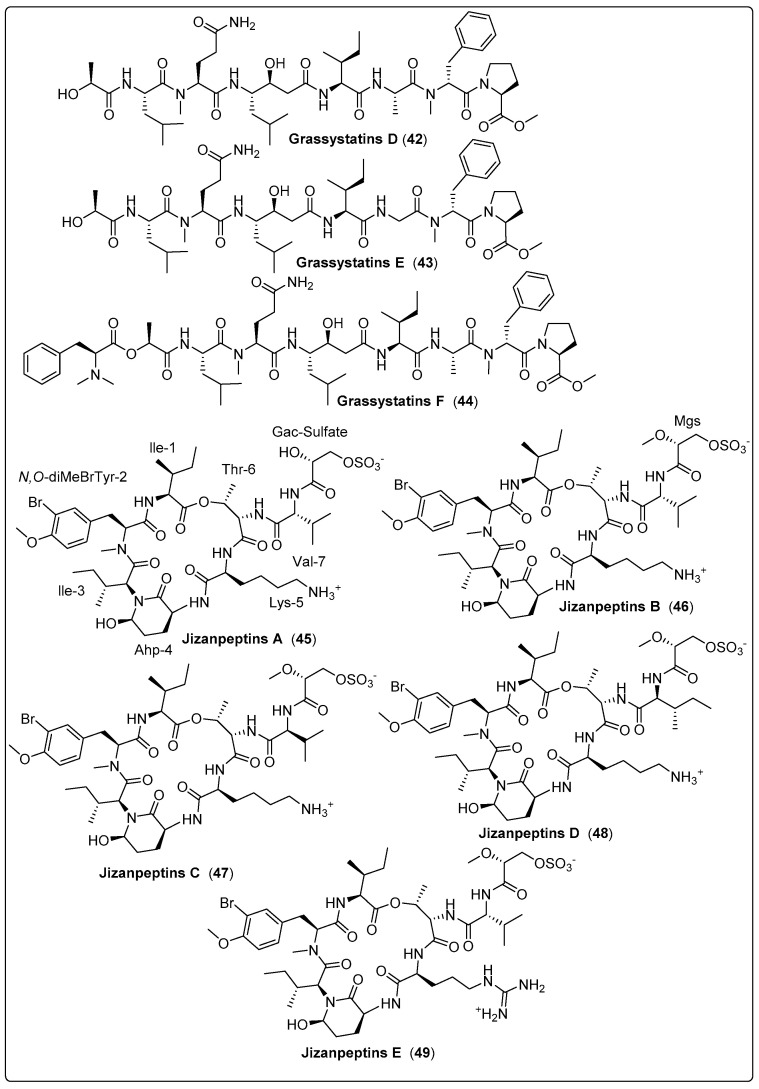

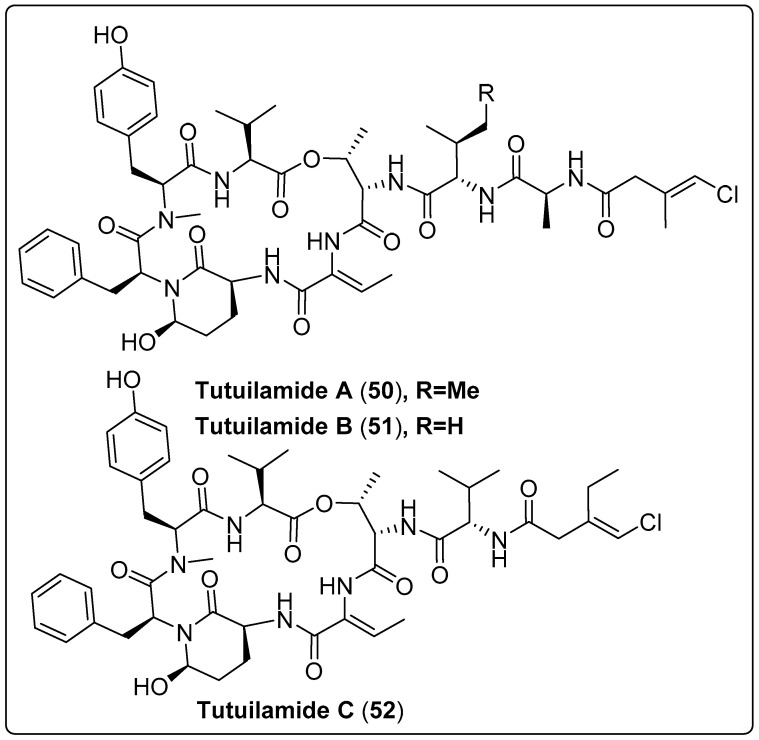
Structures of isolated cyanobacterial compounds from 2017 to 2022 with serine proteases inhibitory effect.

Three bioactive compounds, grassystatin D and F (**42**–**44**), were isolated from marine cyanobacteria coded with VPG 14-61. Three compounds were tested for protease inhibitory activity using cathepsin D and E. The findings revealed that compound (**44**) exhibits a potent effect with IC_50_ values of 50 nM and 0.5 nM for cathepsin D and E, respectively. The mechanism of action occurs by inhibiting cleavage of cystatin C and PAI-1 [61]. Gallegos and his colleagues isolated five bioactive compounds named Jizanpeptin A-E (**45**–**49**) from marine cyanobacteria *Symploca* sp. The five bioactive compounds were tested for inhibition of serine protease activity using trypsin and chymotrypsin. The most potent bioactive compound (**47**) exhibits activity with IC_50_ 72 ± 17 nM and 1400 ± 700 nM for trypsin and chymotrypsin, respectively. The cytotoxicity of the five compounds was also assessed using HeLa cervical and NCI-H460 lung cancer. The findings recorded the absence of any significant cytotoxicity to two human cancer cell types [62]. Another three cyclic peptide bioactive compounds, namely tutuilamides A–C (**50**–**52**), were isolated from *Schizothrix* sp. and *Coleofasciculus* sp. by Keller and co-authors (2020). These compounds were tested as protolytic inhibitors for the S1 family (elastase, chymotrypsin, trypsin) and the S8 family (proteinase K) of serine proteases. The most dominant inhibitors of elastase were (**50** and **51**), with IC_50_ in the 1–2.05 nM range. Regarding chymotrypsin, the potency of (**50–52**) presented IC_50_ values ranging from 542 nM to 1014 nM. As expected, no inhibition of trypsin was detected, while compounds (**50** and **51**) showed significant activity for proteinase K inhibition, with IC_50_ values of 103.7 and 87.6 nM, respectively. The mode of action was explained as binding to active sites in a substrate-like manner. Cytotoxicity was also tested using the H-460 human lung cancer cell line as a model. IC_50_ values of the compounds ranged between 0.53–4.78 µM. The only flaw in this study was the absence of positive or negative controls [63]. We recommend the most bioactive compounds that screened in Table 3, Scheme 3 and their spectral analysis reviewed in Table S3 need further assessment and are considered promising leads for infectious diseases.

**Table 3:** Screening for bioactive compound serine protease inhibition targets isolated from marine cyanobacteria between 2017 and 2022.

Compound Name/Class	Source/Place and Date	Separation Tools	Pharmacological Activity	Reference
Grassystatin D, peptide (**42**)	VPG 14-61/Cetti Bay, Guam (June 2014)	SPE and RP-HPLC	Protease inhibitory activity Assays: Cell viability and inhibition assay Model: Breast cancer cell line (MDA-MB-231) Cathepsin D IC_50_: 2000 nM Cathepsin E IC_50_: 30 nM NC: DMSO	[61]
Grassystatin E, peptide (**43**)	VPG 14-61/Cetti Bay, Guam (June 2014)	SPE and RP-HPLC	Protease inhibitory activity Assays: Cell viability and inhibition assay Model: Breast cancer cell line (MDA-MB-231) Cathepsin D IC_50_: 900 nM Cathepsin E IC_50_: 5 nM NC: DMSO	[61]
Grassystatin F, peptide (**44**)	VPG 14-61/Cetti Bay, Guam (June 2014)	SPE and RP-HPLC	Protease inhibitory activity Assays: Cell viability and inhibition assay Model:Breast cancer cell line (MDA-MB-231) Cathepsin D IC_50_: 50 nM Ccathepsin E IC_50_: 0.5 nM NC: DMSO	[61]
Jizanpeptin A, depsipeptide (**45**)	*Symploca* sp./Red Sea, coast of Jizan, Saudi Arabia (2013)	HPLC, NP-VLC, and SPE	Inhibitory activity of Serine Proteases Such as: Trypsin IC_50_: 160 ± 30 nM Chymotrypsin IC_50_: >10,000 nM	[62]
Jizanpeptin B, depsipeptide (**46**)	*Symploca* sp./Red Sea, coast of Jizan, Saudi Arabia (2013)	HPLC, NP-VLC, and SPE	Inhibitory activity of Serine Proteases Such as: Trypsin IC_50_: 190 ± 20 nM Chymotrypsin IC_50_: >10,000 nM	[62]
Jizanpeptin C, depsipeptide (**47**)	*Symploca* sp./Red Sea, coast of Jizan, Saudi Arabia (2013)	HPLC, NP-VLC, and SPE	Inhibitory activity of Serine Proteases Such as: Trypsin IC_50_: 72 ± 17 nM Chymotrypsin IC_50_: 1400 ± 700 nM	[62]
Jizanpeptin D, depsipeptide (**48**)	*Symploca* sp./Red Sea, coast of Jizan, Saudi Arabia (2013)	HPLC, NP-VLC, and SPE	Inhibitory activity of Serine Proteases Such as: Trypsin IC_50_: 1000 ± 250 nM Chymotrypsin IC_50_: >10,000 nM	[62]
Jizanpeptin E, depsipeptide (**49**)	*Symploca* sp./Red Sea, coast of Jizan, Saudi Arabia (2013)	HPLC, NP-VLC, and SPE	Inhibitory activity of Serine Proteases Such as: Trypsin IC_50_: 150 ± 20 nM Chymotrypsin IC_50_: >10,000 nM	[62]
Tutuilamide A, cyclic peptide (**50**)	*Schizothrix* sp./Island of Tutuila in American Samoa (2016)	HPLC, VLC, and SPE	Inhibitory activity of Serine Proteases Such as: Elastase IC_50_: 1.18 nM Chymotrypsin IC_50_: 1014 nM Trypsin IC_50_: >20,000 nM Proteinase K IC_50_: 103.7 nM	[63]
Tutuilamides B, cyclic peptide (**51**)	*Schizothrix* sp./Island of Tutuila in American Samoa (2016)	HPLC, VLC, and SPE	Inhibitory activity of Serine Proteases Such as: Elastase IC_50_: 2.05 nM Chymotrypsin IC_50_: 576.6 nM Trypsin IC_50_: >20,000 nM Proteinase K IC_50_ = 87.6 nM	[63]
Tutuilamide C, cyclic peptide (**52**)	*Coleofasciculus* sp./Island of Tutuila in American Samoa (2016)	HPLC, VLC, and SPE	Inhibitory activity of Serine Proteases Such as: Elastase IC_50_: 4.93 nM Chymotrypsin IC_50_: 542.0 nM Trypsin IC_50_: >20,000 nM Proteinase K IC_50_: 5000 nM	[63]

### 3.4. Antiproliferative and Anticancer Activity

**Scheme 4:**
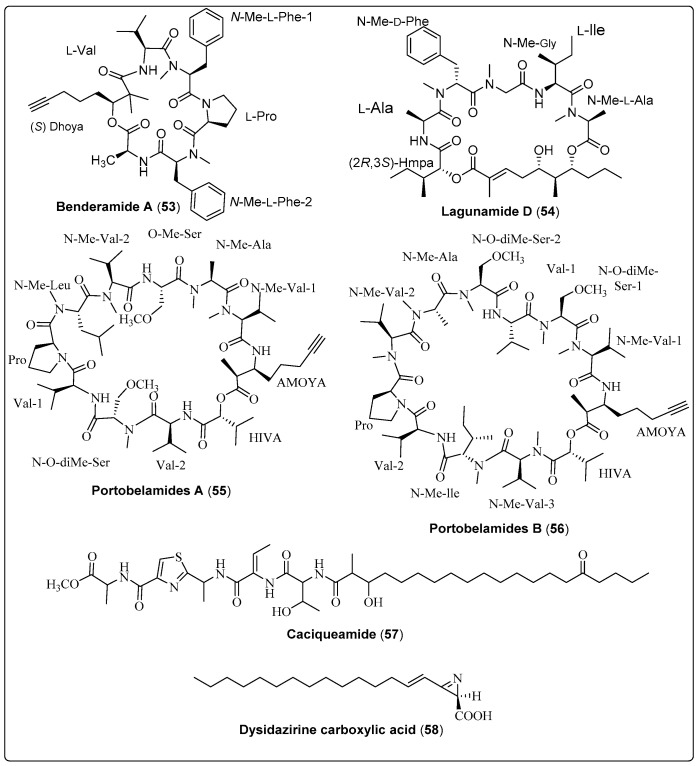
Structures of isolated cyanobacterial compounds from 2017 to 2022 with antiproliferative and anticancer activity.

Lagunamide D (**54**), a natural macrocyclic depsipeptide, was isolated from a collection of marine cyanobacteria (*Dichothrix* sp., *Lyngbya* sp., *Ceramium* sp., and *Rivularia* sp.). It showed significant antiproliferative activity against human lung adenocarcinoma using A549 as an in-vitro model. Compound (**54**) exhibited IC_50_ values of 7.1 ± 1.7 nM. The mechanism of action needs further investigation [64]. Another study reported two secondary metabolites: Portobelamide A and B (**55** and **56**), purified from the marine cyanobacteria *Caldora* sp. Two compounds were evaluated as anticancer using H-460 cells. The findings of compound (**55**) showed 33% survival at a concentration of 0.9 μM [23]. Another carboxylic acid named Dysidazirine carboxylic acid (**58**) was isolated from marine cyanobacteria *Caldora* sp. by Gunasekera and co-authors (2022). The anticancer activity was evaluated against human colon cancer cells (HCT116) in-vitro. Compound (**58**) shows an acceptable effect with IC_50_ values of 79.7 μM, in comparison with the positive control (Gatorbulin-1 with IC_50_ values of 0.8 μM) [25]. Finally, we conclude that in-deep studies require potent bioactive compounds shown in Table 4, structured in Scheme 4 and their spectral analysis reviewed in Table S4 that may be lead compounds for anticancer drugs.

**Table 4:** Screening for bioactive compounds has antiproliferation and anticancer targets isolated from marine cyanobacteria between 2017 and 2022.

Compound Name/Class	Source/Place and Date	Separation Tools	Pharmacological Activity	Reference
Benderamide A, cyclic depsipeptide (**53**)	*Lyngbya* sp./John’s Island, Singapore (June 2016)	HPLC and VFC	Antiproliferative activity Model: MCF-7 breast and PA1 ovarian cancer cell lines NC: DMSO PC: Nutlin Concentration of PC: 10 µM	[65]
Lagunamide D, macrocyclic depsipeptide (**54**)	Collection of marine cyanobacteria (*Dichothrix* sp., *Lyngbya* sp., *Ceramium* sp. and *Rivularia* sp.)/Loggerhead Key in the Dry Tortugas, Florida (May 2015)	HPLC, FCL and SPE	Antiproliferative activity Model: Human lung adenocarcinoma cells (A549) Assay: MTT assay IC_50_: 7.1 ± 1.7 nM NC: cells + medium + solvent control	[64]
Portobelamide A, cyclic depsipeptide (**55**)	*Caldora* sp./Portobelo, Panama: (June 2012)	VLC and HPLC	Anticancer activity Model: Human lung Cancer Cell (H-460 cells) Cytotoxicity: (33% survival at 0.9 μM) PC: (doxorubicin) NC: (DMSO in RPMI 1640 medium)	[23]
Portobelamide B, cyclic depsipeptide (**56**)	*Caldora* sp./Portobelo, Panama: (June 2012)	VLC and HPLC	Anticancer activity Model: Human lung Cancer Cell (H-460 cells) Cytotoxicity: Not cytotoxic PC: (doxorubicin) NC: (DMSO in RPMI 1640 medium)	[23]
Caciqueamide, long chain lipopeptide (**57**)	*Caldora* sp./Portobelo, Panama (June 2012)	VLC and HPLC	Anticancer activity Model: Human lung Cancer Cell (H-460 cells) Cytotoxicity: Not cytotoxic PC: (doxorubicin) NC: (DMSO in RPMI 1640 medium)	[23]
Dysidazirine carboxylic acid, carboxylic acid (**58**)	*Caldora* sp./Fort Lauderdale, Florida (July 2017)	RP-HPLC	Anticancer activity Assay: Cell Viability Model: Human colon cancer cells (HCT116) IC_50_: 79.7 µM Positive control: Gatorbulin-1 IC_50_ for PC: 0.80 µM NC: 0.5% DMSO	[25]

### 3.5. Anti-Quorum Sensing Activity

**Scheme 5:**
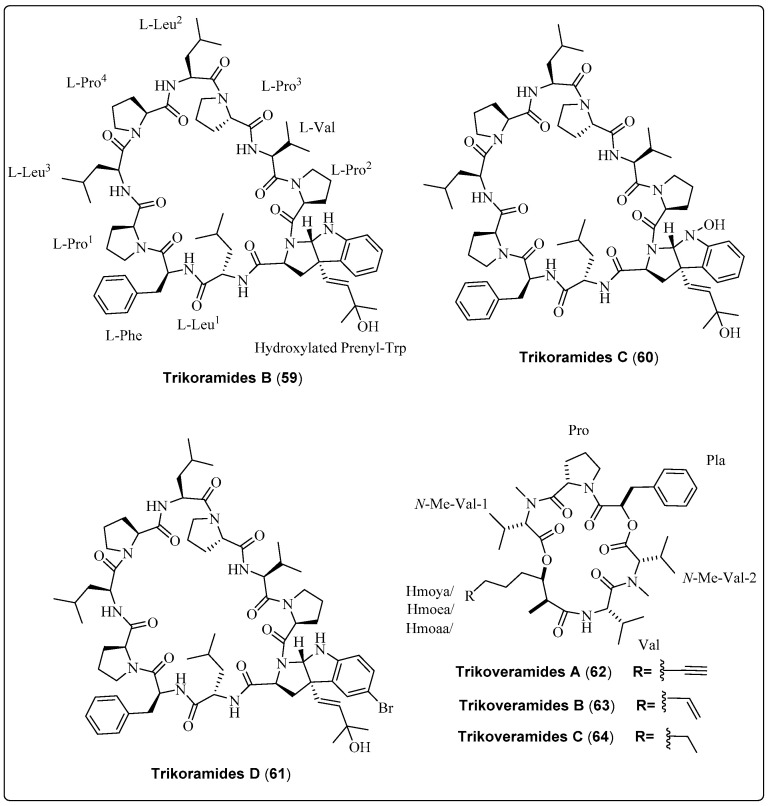
Structures of isolated cyanobacterial compounds from 2017 to 2022 with anti-quorum-sensing activity.

Three bioactive compounds, Trikoramide (B–D) (**59**–**61**), extracted from the marine cyanobacteria *Symploca hydnoides*, were evaluated for their potential as anti-quorum-sensing activity. Compound (**61**) exhibited a significant effect against *Pseudomonas aeruginosa* PAO1 lasB-gfp and rhlA-gfp strains, with IC_50_ values of 19.6 µM and 7.3 µM, respectively. Cytotoxicity of three compounds was assessed using the Acute Lymphoblastic Leukemia Cell Line. Compound (**61**) exhibited a significant effect, with IC_50_ values of 4.7 µM [66]. Another three bioactive compounds, Trikoveramide (A–C) (**62**–**64**), extracted from the marine cyanobacteria *Symploca hydnoides*, were evaluated for their potential as anti-quorum-sensing activity. Compound (**62**) exhibited a significant effect against *Pseudomonas aeruginosa* PAO1 lasB-gfp and rhlA-gfp strains, with an inhibition value of 8%. The cytotoxicity of three compounds was assessed against the MOLT-4 human leukemia cell line. Compound (**63**) exhibited a significant effect, with IC_50_ values of 9.32 µM [67]. We recommend that the researcher investigate for the anti-quorum-sensing activity of marine cyanobacteria bioactive compounds as screened in Table 5, Scheme 5 and their spectral analysis reviewed in Table S5 that may be a lead compound.

**Table 5:** Screening for bioactive compounds has anti-quorum-sensing activity isolated from marine cyanobacteria between 2017 and 2022.

Compound Name/Class	Source/Place and Date	Separation Tools	Pharmacological Activity	Reference
Trikoramide B, decapeptide (**59**)	*Symploca hydnoides*/Trikora beach, Bintan Island (April 2018)	RP-HPLC, VLC	**Anti-quorum-sensing activity** Model: *Pseudomonas aeruginosa* PAO1 *lasB-gfp* and *rhlA-gfp* strains Assay: Anti-quorum-sensing assay IC_50_ for (*lasB-gfp*): No dose-dependent response observed IC_50_ for (*rhlA-gfp*): No dose-dependent response observed	[66]
Trikoramide C, decapeptide (**60**)	*Symploca hydnoides*/Trikora beach, Bintan Island (April 2018)	RP-HPLC, VLC	**Anti-quorum-sensing activity** Model: *Pseudomonas aeruginosa* PAO1 *lasB-gfp* and *rhlA-gfp* strains Assay: Anti-quorum-sensing assay IC_50_ for (*lasB-gfp*): No dose-dependent response observed IC_50_ for (*rhlA-gfp*): No dose-dependent response observed	[66]
Trikoramide D, decapeptide (**61**)	*Symploca hydnoides*/Trikora beach, Bintan Island (April 2018)	RP-HPLC, VLC	**Anti-quorum-sensing activity** Model: *Pseudomonas aeruginosa* PAO1 *lasB-gfp* and *rhlA-gfp* strains Assay: Anti-quorum-sensing assay IC_50_ for (*lasB-gfp*): 19.6 µM IC_50_ for (*rhlA-gfp*): 7.3 µM	[66]
Trikoveramide A, cyclic depsipeptide (**62**)	*Symploca hydnoides*/Trikora beach, Bintan Island (April 2018)	NP-VLC, RP-HPLC	**Anti-quorum-sensing activity** Model: *Pseudomonas aeruginosa* strain PAO1-*lasB-gfp* Assay: Anti-quorum-sensing assay Inhibition: 8%	[67]
Trikoveramide B, cyclic depsipeptide (**63**)	*Symploca hydnoides*/Trikora beach, Bintan Island (April 2018)	NP-VLC, RP-HPLC	**Anti-quorum-sensing activity** Model: *Pseudomonas aeruginosa* strain PAO1-*lasB-gfp* Assay: Anti-quorum-sensing assay Inhibition: 26%	[67]
Trikoveramide C, cyclic depsipeptide (**64**)	*Symploca hydnoides*/Trikora beach, Bintan Island (April 2018)	NP-VLC, RP-HPLC	**Anti-quorum-sensing activity** Model: *Pseudomonas aeruginosa* strain PAO1-*lasB-gfp* Assay: Anti-quorum-sensing assay Inhibition: 45%	[67]

### 3.6. Antibacterial Activity

**Scheme 6:**
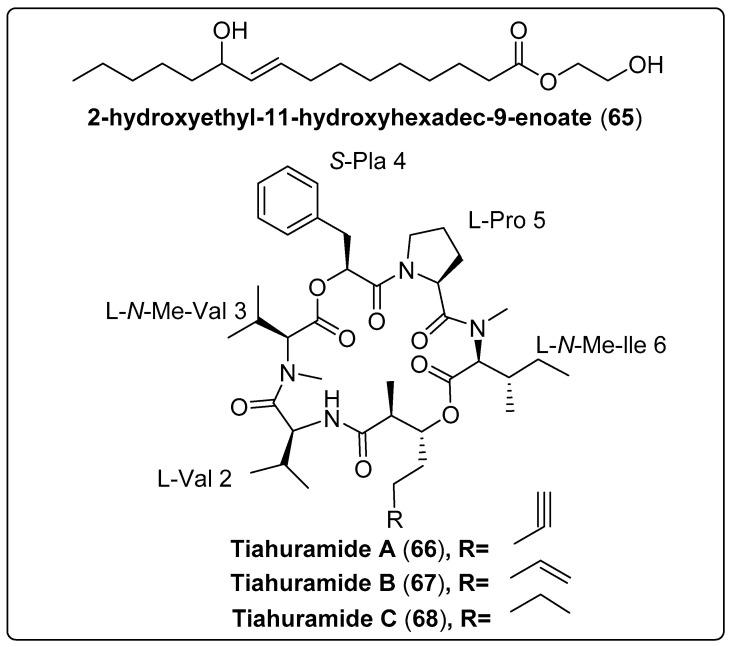
Structures of isolated cyanobacterial compounds from 2017 to 2022 with antibacterial activity.

The bioactive compound, namely 2-hydroxyethyl-11-hydroxyhexadec-9-enoate (**65**) isolated from marine cyanobacteria *Leptolyngbya* sp. The compound was evaluated as an antibacterial activity using *Vibrio harveyi* and *V. parahaemolyticus* as a model. The results reported that compounds exhibit a potent effect with MIC 250–1000 µg/mL^−1^ and 350–1000 µg/mL^−1^, respectively, compared with the positive control (ampicillin; MIC 500 µg/mL^−1^) [68]. Levert et al. (2018) isolated three bioactive compounds: Tiahuramides A–C (**66**–**68**) from marine cyanobacteria *Lyngbya majuscule*. The findings showed that compound (**68**) has potent effects against *Aeromonas salmonicida* with a MIC value of 6.7 µM. The mechanism of action moderately inhibited bacterial growth. The same compound exhibits a significant cytotoxic effect against human neuroblastoma (SH-SY5Y) with an IC_50_ value of 6.0 ± 2.7 µM [69]. We recommend more investigation for bioactive compounds shown in Table 6, structured in Scheme 6 and their spectral analysis reviewed in Table S6 with a potent effect for new lead compounds as antibacterial activity.

**Table 6:** Screening for bioactive compounds have antibacterial activity isolated from marine cyanobacteria between 2017 and 2022.

Compound Name/Compound Description	Source/Place and Date	Separation Tools	Pharmacological Activity	Reference
2-hydroxyethyl-11-hydroxyhexadec-9-enoate, not reported (**65**)	*Leptolyngbya* sp./Gulf of Thailand, Chumpon and Chonburi provinces	CC	Antibacterial activity Models: *Vibrio harveyi* and *V. parahaemolyticus* Assay: agar plate diffusion Concentrations: 0, 100, 125, 250, 350, 500, 650, 800 and 1000 µg/mL^−1^ MIC: 250–1000 µg/mL^−1^ for *V. harveyi* MIC: 350–1000 µg/mL^−1^ for *V. parahaemolyticus* PC: ampicillin (500 µg/mL^−1^) NC: penicillin (800 µg/mL^−1^)	[68]
Tiahuramide A, cyclic depsipeptide (**66**)	*Lyngbya* *majuscule* Harvey ex Gomont/Tiahura sector, Moorea Island in French Polynesia	TLC and HPLC	Antibacterial activity Assay: 96-well plates Model: *Aeromonas salmonicida* (MIC: 27 µM) *Vibrio anguillarum* (MIC: 33 µM) *Shewanella baltica* (MIC: >50 µM) *Escherichia coli* (MIC: 35 µM) *Micrococcus luteus* (MIC: 47 µM)	[69]
Tiahuramide B, cyclic depsipeptide (**67**)	*Lyngbya* *majuscule* Harvey ex Gomont/Tiahura sector, Moorea Island in French Polynesia	TLC and HPLC	Antibacterial activity Assay: 96-well plates Model: *Aeromonas salmonicida* (MIC: 9.4 µM) *Vibrio anguillarum* (MIC: 8.5 µM) *Shewanella baltica* (MIC: 22 µM) *Escherichia coli* (MIC: 12 µM) *Micrococcus luteus* (MIC: 29 µM)	[69]
Tiahuramide C, cyclic depsipeptide (**68**)	*Lyngbya* *majuscule* Harvey ex Gomont/Tiahura sector, Moorea Island in French Polynesia	TLC and HPLC	Antibacterial activity Assay: 96-well plates Model: *Aeromonas salmonicida* (MIC: 6.7 µM) *Vibrio anguillarum* (MIC: 7.4 µM) *Shewanella baltica* (MIC: 16 µM) *Escherichia coli* (MIC: 14 µM) *Micrococcus luteus* (MIC: 17 µM)	[69]

### 3.7. Other Activities

**Scheme 7:**
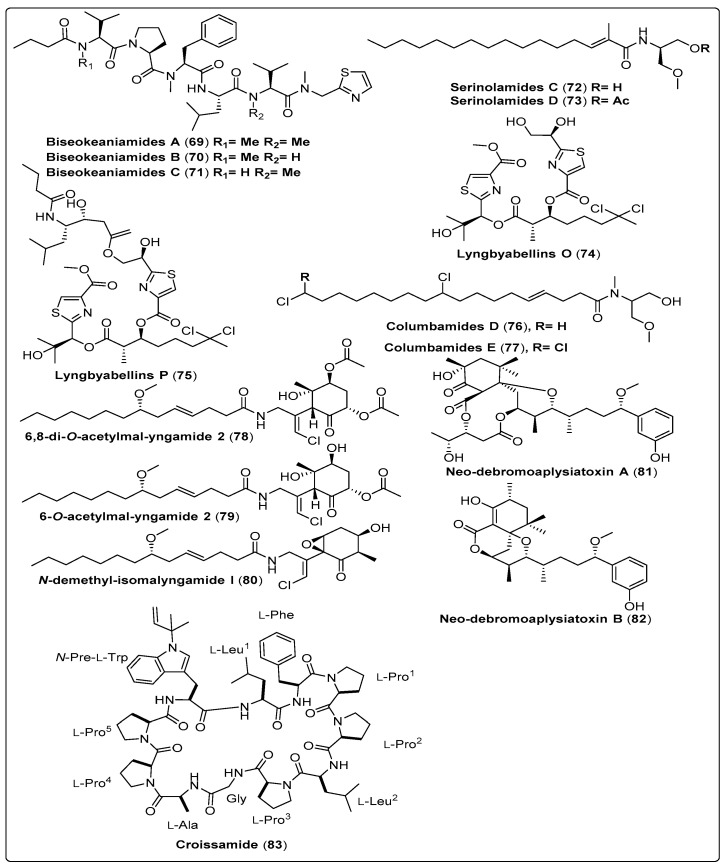
Structures of isolated cyanobacterial compounds from 2017 to 2022 with different activities.

Iwasaki and his colleagues isolated three bioactive compounds named Biseokeaniamide A–C (**69**–**71**) from marine cyanobacteria *Okeania* sp. Sterol *O*-acyltransferase inhibitory activity was assessed for three compounds using two different assays. This compound (**69**) showed a potent effect regarding cell-based assay with IC_50_ values 1.8 µM for SOAT1 and IC_50_ 1.3 µM for SOAT2. While regarding Enzyme-based assay shows IC_50_ values 1.8 µM for SOAT1 and 9.6 µM for SOAT2. Cell growth inhibition was assessed for three compounds. Compound (**70**) shows a potent effect with IC_50_ value of 4.5 µM for HeLa cells and 19 µM for HL60 cells [70]. Another two bioactive compounds, Lyngbyabellin O and B (**74** and **75**), were isolated from marine cyanobacteria *Okeania* sp. as shown in Table 7, structured in Scheme 7 and their spectral analysis reviewed in Table S7. In-vitro, a study showed significant antifouling activity against *Amphibalanus amphitrite* larvae. The findings reported that (**74** and **75**) exhibited high EC_50_ values of 0.38 μM and 0.73 μM, respectively [71]. Han et al. (2018) isolated two bioactive compounds named Neo-debromoaplysiatoxin A and B (**81**–**82**) from marine cyanobacteria *Lyngbya* sp. The blocking activity against Kv1.5 was evaluated for two compounds using a Chinese hamster ovary. The two compounds exhibit a highly potent effect, with IC_50_ values of 6.94 ± 0.26 µM and 0.30 ± 0.05 µM, respectively. The mechanism of action displayed provides modulating ionic channels, and links between the DAT target, protein kinase C, and cell regulation [72].

**Table 7:** Screening for bioactive compounds has different activity isolated from marine cyanobacteria between 2017 and 2022.

Compound Name/Class	Source/Place and Date	Separation Tools	Pharmacological Activity	Reference
Biseokeaniamide A, linear lipopeptide (**69**)	*Okeania* sp./Bise, Okinawa Prefecture, Japan (April 2015)	RP-HPLC and OSD-CC	Cell growth-inhibitory activity Model: HeLa and HL60 cells Assay: MTT assay IC_50_: 29 µM for HeLa cells IC_50_: 30 µM for HL60 cells PC: Tunicamycin Sterol *O*-acyltransferase inhibitory activity Model: CHO cells of African green monkey Assays: Cell-based assay IC_50_: 1.8 µM for SOAT1 PC: Purpactin A (IC_50_: 2.5 µM for SOAT1) IC_50_: 1.3 µM for SOAT2 PC: Purpactin A (IC_50_: 1.5 µM for SOAT2) Enzyme-based assay IC_50_: 1.8 µM for SOAT1 PC: Purpactin A (IC_50_: 0.9 µM for SOAT1) IC_50_: 9.6 µM for SOAT2 PC: Purpactin A (IC_50_: 1.8 µM for SOAT2)	[70]
Biseokeaniamide B, linear lipopeptide (**70**)	*Okeania* sp./Bise, Okinawa Prefecture, Japan (April 2015)	RP-HPLC and OSD-CC	Cell growth-inhibitory activity Model: HeLa and HL60 cells Assay: MTT assay IC_50_: 4.5 µM for HeLa cells IC_50_: 19 µM for HL60 cells PC: Tunicamycin Sterol *O*-acyltransferase inhibitory activity Model: CHO cells of African green monkey Assays: Cell-based assay IC_50_: 6.9 µM for SOAT1 PC: Purpactin A (IC_50_: 2.5 µM for SOAT1) IC_50_: 2.5 µM for SOAT2 PC: Purpactin A (IC_50_: 1.5 µM for SOAT2) Enzyme-based assay IC_50_: 6.8 µM for SOAT1 PC: Purpactin A (IC_50_: 0.9 µM for SOAT1) IC_50_: 9.9 µM for SOAT2 PC: Purpactin A (IC_50_: 1.8 µM for SOAT2)	[70]
Biseokeaniamide C, linear lipopeptides (**71**)	*Okeania* sp./Bise, Okinawa Prefecture, Japan (April 2015)	RP-HPLC and OSD-CC	Cell growth-inhibitory activities Model: HeLa and HL60 cells Assay: MTT assay IC_50_: 43 µM for HeLa cells IC_50_: >100 µM for HL60 cells PC: Tunicamycin Sterol *O*-acyltransferase inhibitory activity Model: CHO cells of African green monkey Assays: Cell-based assay IC_50_: >12 µM for SOAT1 PC: Purpactin A (IC_50_: 2.5 µM for SOAT1) IC_50_: 9.6 µM for SOAT2 PC: Purpactin A (IC_50_: 1.5 µM for SOAT2) Enzyme-based assay IC_50_: 11 µM for SOAT1 PC: Purpactin A (IC_50_: 0.9 µM for SOAT1) IC_50_: >32 µM for SOAT2 PC: Purpactin A (IC_50_: 1.8 µM for SOAT2)	[70]
Serinolamide C, fatty acid amide (**72**)	*Okeania* sp./Jeddah, Saudi Arabia (April 2015)	RP-HPLC	Antifouling activity Model: *Amphibalanus amphitrite* larvae EC_50_: Not reported Cytotoxic activity Model: Breast cancer cells (MCF7) GI_50_: Not reported	[71]
Serinolamide D, fatty acid amide (**73**)	*Okeania* sp./Jeddah, Saudi Arabia (April 2015)	RP-HPLC	Antifouling activity Model: *Amphibalanus amphitrite* larvae EC_50_: Not reported Cytotoxic activity Model: Breast cancer cells (MCF7) GI_50_: Not reported	[71]
Lyngbyabellin O, Not reported (**74**)	*Okeania* sp./Jeddah, Saudi Arabia (April 2015)	RP-HPLC	Antifouling activity Model: *Amphibalanus amphitrite* larvae EC_50_: 0.38 µM Cytotoxic activity Model: Breast cancer cells (MCF7) GI_50_: >160 µM	[71]
Lyngbyabellin P, Not reported (**75**)	*Okeania* sp./Jeddah, Saudi Arabia (April 2015)	RP-HPLC	Antifouling activity Model: *Amphibalanus amphitrite* larvae EC_50_: 0.73 µM Cytotoxic activity Model: Breast cancer cells (MCF7) GI_50_: 9 mM	[71]
Columbamide D, chlorinated fatty acid amide (**76**)	*Moorea bouillonii*/Mantanani Island in Sabah, Malaysia	OSD-CC and HPLC	The biological activity could not be assessed because of highly cytotoxictrace amounts	[73]
Columbamide E, chlorinated fatty acid amide (**77**)	*Moorea bouillonii*/Mantanani Island in Sabah, Malaysia	OSD-CC and HPLC	The biological activity could not be assessed because of highly cytotoxic trace amounts	[73]
6,8-di-*O*-acetylmal-yngamide 2, Malyngamide serie (**78**)	*Moorea producens*/Bise, Okinawa Prefecture, Japan (April 2016)	OSD-CC and HPLC	Stimulation of glucose uptake Model: L6 myotubes Concentration: (10–40 µM) PC: Nepodin NC: DMSO	[74]
6-*O*-acetylmal-yngamide 2, Malyngamide series (**79**)	*Moorea producens*/Bise, Okinawa Prefecture, Japan (April 2016)	OSD-CC and HPLC	Stimulation of glucose uptake Model: L6 myotubes Concentration: (10–40 µM) PC: Nepodin NC: DMSO	[74]
*N*-demethyl-isomal-yngamide I, Malyngamide series (**80**)	*Moorea producens*/Bise, Okinawa Prefecture, Japan (April 2016)	OSD-CC and HPLC	Stimulation of glucose uptake Model: L6 myotubes Concentration: (10–40 µM) PC: Nepodin NC: DMSO	[74]
Neo-debromoaplysiatoxin A, polyketide (**81**)	*Lyngbya* sp./Hainan Island, China (November of 2016)	UPLC and HPLC	Blocking activity against Kv1.5 Model: Chinese hamster ovary IC_50_ = 6.94 ± 0.26 µM PC: Not reported	[72]
Neo-debromoaplysiatoxin B, polyketide (**82**)	*Lyngbya* sp./Hainan Island, China (November of 2016)	UPLC and HPLC	Blocking activity against Kv1.5 Model: Chinese hamster ovary IC_50_ = 0.30 ± 0.05 µM PC: Not reported	[72]
Croissamide, cyclic peptide (**83**)	*Symploca* sp./Minna Island, Okinawa	RP-HPLC and RP-CC	Did not show any significant activities for antimalarial activity, protease inhibitory activity and antibacterial activity	[75]

Cyanobacteria are prolific bioactive secondary metabolite producers. These bioactive compounds fall under the main classes i.e., alkaloids, peptides, linear-, cyclic peptides, depsipeptides, and fatty acid amides. Some bioactive compounds identified from freshwater, marine, and terrestrial cyanobacteria share similarities in the exoskeleton. For example, dragonamides a group of linear peptides isolated from freshwater cyanobacteria *Lyngbya aestuarii* share similarities of exoskeleton and pharmacological effects as antiparasitic activity with linear peptides iheyamides isolated from marine cyanobacteria *Dapis* sp. [58,76]. Another example of similarities is a cyclic depsipeptide named nostopeptins A isolated from cultured freshwater cyanobacteria *Nostoc minutum* and dudawalamides a group of cyclic depsipeptides isolated from marine cyanobacteria *Moorea producens* [28,76]. A group of macrocyclic depsipeptides for example microviridin isolated from the freshwater cyanobacteria, *Planktothrix agardhii*, and *Nostoc minutum* collected from several geographical regions, has similarity in skeleton with lagunamide D isolated from collection of marine cyanobacteria (*Dichothrix* sp., *Lyngbya* sp., *Ceramium* sp., and *Rivularia* sp.) [64,76]. Finally, we conclude that fresh, marine and terrestrial cyanobacteria share some of similarities in the exoskeleton which lead to similarities in the pharmacological targets.

## 4. Marine Cyanobacteria in Clinical Trials

Recently, marine cyanobacteria have passed into the various phases of clinical trials, and some are still under study, as shown in Table 8. For example, Apomivir^®^ extracted from marine cyanobacteria, passed into phase 2 clinical trials. Based on preclinical studies it shows antiviral activity, especially for seasonal influenza viruses (In-fluenza virus A and B). Apomivir^®^, is being tested for treating influenza, with a placebo as a control. It can be administered as a capsule with a dosage of 1 capsule twice daily for 5 days. The age group of the study ranges between 20 years to 65 years (https://clinicaltrials.gov (accessed on 15 April 2023), NCT01677689). Another example, water-soluble Extract of the cyanobacteria *Aphanizomenon* Flos-aquae (AFA), under assessing the Congestive Heart Failure Chronic. Doses were administered orally: 2 capsules 3 time a day. The findings reporting that the improvement in patient’s quality of life after 12 months of trial admission (https://clinicaltrials.gov (accessed on 15 April 2023), NCT04515537).

## 5. Chemical Property Space

**Figure 4:**
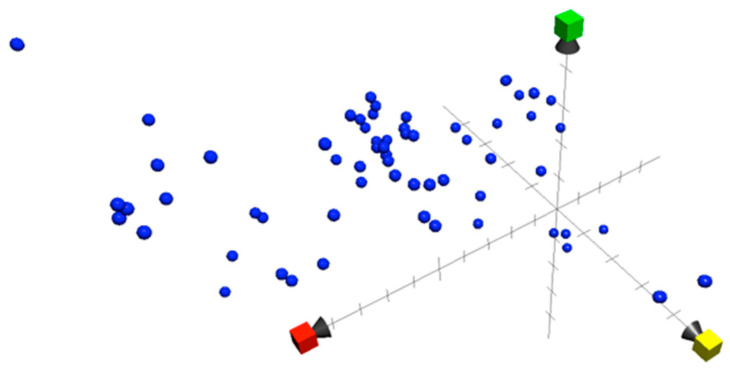
Chemical property Space for cyanobacterial bioactive compounds constructed in three dimensions: PC1 (x = red), PC2 (y = yellow), and PC3 (green).

The introduction of new computational tools supports scientists in the precise and detailed investigation of data [77]. The chemical property of all the bioactive compounds in this review was investigated using the Chemical Global Positioning System—Natural product Web Chem GPS-NP Web (http://chemgps.bmc.uu.se (accessed on 2 April 2023). Analysis of 35+ physiochemical properties resulted in the identification of eight principal components [78]. The three-dimensional chemical property space was constructed using the first three principal components for each compound, as shown in Figure 4. The advantage of using these three components is that they provide maximum variations among the available eight components. The bioactive compounds were spread across the first dimension, indicating a wide-ranging distribution in molecular weight, shape, and size (X axis in red; Y axis in yellow and Z axis in green), but with the majority of compounds lying right across the middle of the range. Two compounds (Bromoiesol A and Bromoiesol B) showed unevenness in the second dimension, indicating unique aromatic and conjugation-related properties, while four compounds (Dragocin derivatives) showed disproportional opposite positioning on the third dimension, describing lipophilicity, polarity, and H-bond capacity. Crossing the cyanobacterial natural compound chemical property space with the bioactive compound cyanobacterial chemical property space presented in this review confirms the unique space occupied by cyanobacterial bioactive compounds. The cyanobacterial compound chemical property space provides a comprehensive overview of the structural diversity and chemical characteristics of bioactive compounds originating from cyanobacteria. Cyanobacterial bioactive property space is a good tool for fishing (comparison) for similar compounds with similar properties and activities [79,80]. This new approach of exploring the cyanobacterial compounds based on their physicochemical properties and comparing them with other groups of compounds (with certain biological activities) confirms the diversity in compounds and activities. The criteria for similarity to an intended compound or group of compounds from any source can be calculated using Euclidian distance (ED) in eight-dimensional space as follow:

(1) $$d\left(p,q\right)=\sqrt{(p_{1}-q_{1}{)}^{2}+(p_{2}-q_{2}{)}^{2}+\ldots +(p_{i}-q_{i}{)}^{2}+\ldots +(p_{n}-q_{n}{)}^{2}}$$

where *d* (distance) and *p*, *q* (points): points *p* = (*p*_1_, *p*_2_, …, *p_n_*) and *q* = (*q*_1_, *q*_2_, …, *q_n_*) in Euclidean n-space. In this case the shorter is the distance the higher is the similarity.

It aids in revealing novel chemical scaffolds, discovering structure-activity relationships, and expediting the usage of bioactive compounds within therapeutic or biotechnological applications. This strategy can boost the identification of leading compounds with target functions and hold promise for further research and discovery in the field of natural products.

## 6. Biological Properties

### 6.1. Mechanism of Marine Cyanobacteria in Nitrogen Fixation

**Figure 5:**
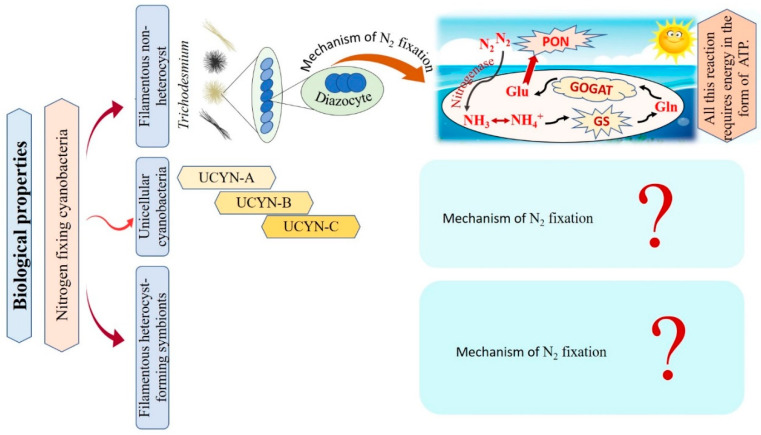
Flowchart for the different types of nitrogen-fixative cyanobacteria with their mechanisms of action.

Cyanobacteria are recognized as the main N_2_-fixing microorganisms in the marine environment that participate in the universal nitrogen cycle [10,81]. N_2_-fixing cyanobacteria are grouped into filamentous non-heterocyst, unicellular cyanobacteria, and filamentous heterocyst-forming symbionts Figure 5 [10]. Regarding filamentous non-heterocyst, the genus *Trichodesmium* represents the most conspicuous marine organisms in terms of N_2_ fixing [82]. They are estimated to be responsible for approximately 50% of the global natural N_2_ fixation. Their nitrogen fixation process occurs during the day at temperatures ranging between 24–30°C, but stops at night due to inactivation and degradation of the nitrogenase enzyme [31,83]. Where N_2_ fixation occurs in differentiated cells of *Trichodesmium* spp., these cells consistently remain in clusters of about 3 to 20 [84]. The N products, such as ammonia (NH_3_), ammonium (NH_4_^+^), or the amino acid glutamine, are directly produced as a result of the reduction of N_2_ [85,86]. The following synthesis of glutamine and glutamate by a glutamine synthetase/glutamine oxoglutarate aminotransferase (GS/GOGAT) reaction also requires energy in the form of 1 ATP and 1 NADPH + H^+^. Thus, *Trichodesmium*’s capacity to fix nitrogen is highly reliant on the bioavailability of energy [87]. Unicellular N_2_-fixing cyanobacteria were first discovered through the amplification of nitrogenase genes and gene transcripts (mRNAs) from oceanic water samples [88]. According to recent studies, unicellular cyanobacteria (UCYN) have a high ability to fix N_2_ within a cell-size fraction below 10 μm. These unicellular cyanobacteria have been classified into three groups based on their *nifH* gene phylogeny: UCYN-A, UCYN-B, and UCYN-C [88,89,90]. In the UCYN-A group, cyanobacterial nitrogenase gene sequences were most tightly linked to sequences from the marine unicellular cyanobacterium *Cyanothece* sp. strain ATCC 51142 [91]. Despite numerous attempts, UCYN-A cyanobacteria have not been successfully cultivated [92]. UCYN-A cyanobacteria have genotypes that have not previously been identified in free-living cyanobacteria, and they lack the genetic ability for oxygenic photosynthesis. Furthermore, nitrogenase genes are most abundant in UCYN-A during the light period, and thus can fix N_2_ during daylight [90]. UCYN-B is a group of free-living unicellular cyanobacteria that fix N_2_ [93]. By studying the geographical populations of these free-living creatures, it may be concluded that they significantly contribute to the global N_2_ fixation process [94]. UCYN-B have only been identified in surface water and at 25% light depths (14 m) and is small in size (<10 μm) [95,96]. They fix N_2_ during the night [10]. In UCYN-B (*Crocosphaera watsonii*), nitrogenase enzyme synthesis begins just before nightfall to prepare for the upcoming N_2_ fixing activity during the night [97]. UCYN-C is a group of unicellular free-living cyanobacteria that fix N_2_ during the night [94,98]. It includes several cultivated cyanobacteria, such as *Cyanothece* sp. strain ATCC51142 and TW3 [99,100]. By 1993, Reddy and colleagues isolated a unicellular cyanobacterium, *Cyanothece* sp. ATCC51142, on the intertidal sands of the Texas Gulf Coast [101]. Their symbiotic associations play vital roles in chemical defense, as well as supplying partners with energy and organic products of carbon or nitrogen fixation [102]. In numerous oceanic diatom taxa, heterocyst-forming cyanobacterial symbionts are frequently observed [103]. In the microenvironment of a photosynthetic symbiotic partner cell, such as diatoms, heterocyst-forming cyanobacteria have a beneficial effect, where the oxygen-sensitive nitrogenase protein is inhibited as a result of O_2_ concentrations [10]. *Richelia intracellularis* J. Schmidt 1901 is a filamentous cyanobacteria frequently found either free-living or in symbiosis with the diatoms *Rhizosolenia* Brightwell 1858 and *Chaetoceros* Ehrenberg 1844 in the plankton of the warm oceans [102]. Cyanobacteria nitrogen fixation has various impacts that can be explained as follows: firstly, cyanobacteria are significant bioavailable nitrogen suppliers to the pelagic and benthic food webs that support fish productivity by fixing dissolved N_2_. The food web benefits from bioavailable nitrogen in addition to the fresh or decaying filamentous cyanobacteria to nourish various invertebrates and release more nitrogen by cyanobacterial cells. Secondly, their ability to get beyond summertime nitrogen limitation by the fixation of dissolved nitrogen. Thirdly, they can contribute to enhancing productivity in different agricultural and ecological situations by building up soil fertility and increasing yield [104,105,106]. We conclude that only filamentous non-heterocyst cases have contributed to the explanation of the mechanism of nitrogen fixation. On the other hand, there are limitations in the data related to unicellular cyanobacteria, and filamentous heterocyst-forming symbionts, as there are no papers explaining the role of the nitrogen fixation mechanism in these cases. Taken together, nitrogen fixation has important impacts in various fields, i.e., agriculture and ecology, reflecting a potential benefit on the economy.

### 6.2. Mechanism of Marine Cyanobacteria in CO_2_ Fixation

**Figure 6:**
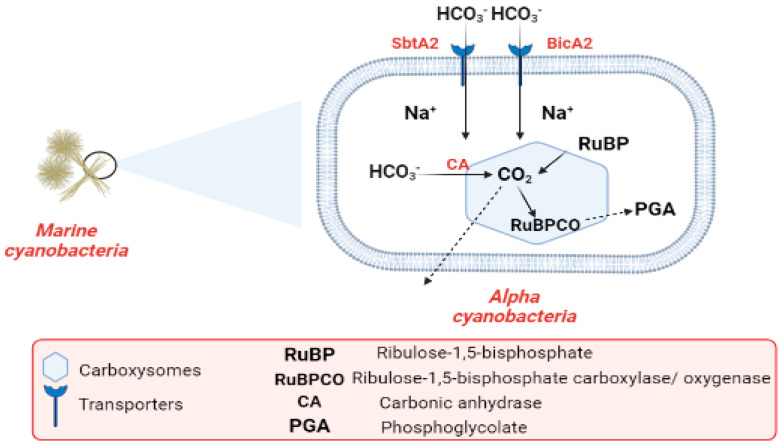
Flow chart explaining the carbon cycle in marine cyanobacteria.

Industrialization and the burning of fossil fuels are to blame for the alarmingly high CO_2_ levels in the atmosphere. CO_2_ concentrations in surface waters have increased with the rise in atmospheric CO_2_ during the past century. Cyanobacteria are one of the most promising organisms for CO_2_ capture [107,108,109]. The most common photoautotrophic lineage on Earth comprises cyanobacteria. Their effectiveness in photoautotrophism relies on a collection of adaptations known as the CO_2_-concentrating mechanism (CCM). The CCM aims to increase the efficiency of CO_2_ fixation by promoting the carboxylase reaction through improving the chemical conditions around the main CO_2_-fixing enzyme, D-ribulose 1,5-bisphosphate carboxylase/oxygenase (RubisCO), and suppressing the oxygenase reaction [110]. In cyanobacteria, RubisCO is enclosed within a class of protein-rich structures known as carboxysomes. The selectively permeable protein shell of the carboxysomes contains the CO_2_-fixation enzyme, as well as the carbonic anhydrase enzyme, which provides CO_2_ from a cytoplasmic bicarbonate pool [110,111]. The carboxysomes have two main types, α-cyanobacteria, which are found in marine water and β-cyanobacteria, present in fresh water [111]. In α-cyanobacteria, the RubisCO is categorized as (RuBisCO Form IA) [112]. In α-cyanobacteria, up to two different types of plasma membrane-associated bicarbonate transporters, SbtA2 (high-affinity Na^+^/HCO_3_^−^) and BicA2 (medium to low-affinity Na^+^/HCO_3_^−^) promote CO_2_ fixation [110]. In the first stage of the (CCM), bicarbonate is concentrated inside the cell via transporters in the cell membrane. In the second stage of (CCM), the carboxysome plays a vital role in the enhancement of CO_2_ fixation by co-localizing the two enzymes (CA) and (RuBisCO). Bicarbonate is assumed to enter the carboxysome through proteinaceous shell pores, and once inside, it is converted to CO_2_ and used by RuBisCO. The conversion of HCO_3_^−^ to CO_2_ is catalyzed by the CA enzyme. After CO_2_ fixation, some of it exits into the cytosol, while some promptly combines with RuBP to form phosphoglycolate Figure 6 [111,113]. The components of the cyanobacterial CO_2_-concentrating mechanism (CCM) have a vital role in the improvement of the efficiency of photosynthetic CO_2_ fixation in the chloroplasts of crop plants via specific cyanobacterial transporters, accordingly leading to better crop yields [114]. Finally, we conclude that the importance of CO_2_ revolves around the conversion of inorganic carbon to organic carbon through vital processes. 

## 7. Effect of Climate Changes on Marine Cyanobacteria

Planet Earth and its aquatic ecosystems are being drastically altered by climate change [115]. Climate changes manifest as rising CO_2_, temperature, and salinization. Cyanobacteria have some features that can adapt to the altered aquatic environmental conditions, i.e., efficient CO_2_ and nutrient uptake mechanisms, and are well protected from light and UV radiation [116]. The rise in atmospheric CO_2_ levels makes marine surface waters more acidic. Levitan et al. (2007) reported that an elevation in CO_2_ concentration was shown to enhance C:N ratios and N_2_ fixation in the open ocean by using the cyanobacterium *Trichodesmium erythraeum* [107,116]. Additionally, a factor contributing to the enhancement of nitrogen fixations is the reduction in O_2_ levels caused by climate change, which is explained by the notion that nitrogenase enzymes responsible for N_2_ fixation are strongly inhibited by oxygen. In agreement and according to the literature, *Trichodesmium* N_2_ fixation takes place during the day along with oxygenic photosynthesis [95]. Similarly, and with high temperatures, some marine cyanobacteria species exhibit optimal growth [117]. Otherwise, the structure of planktonic and benthic microalgal communities is also significantly impacted by rising salinity levels. Some common cyanobacterial species, such as *Anabaena*, *Anabaenopsis*, *Microcystis*, and *Nodularia*, are tolerant to salt. For example, the growth of *Microcystis aeruginosa* is unaffected by 30% salinity in seawater [116,118]. On the other hand, the increase of the vertical density stratification is one impact of salinization, and buoyant CyanoHABs benefit from this stratification, as reported earlier. The existence of CyanoHABs resulting from climate change promotes the turbidity of aquatic environments and hence restricts light penetration into ecosystems, thus being considered an adverse but negligible effect. According to the literature, the creatures most affected by this turbidity are macrophytes and microalgae, where their growth is suppressed somehow [116]. All in all, marine cyanobacteria seem to be one of the lifeguards as they combat the severity of climate change. 

## 8. Conclusions and Future Perspectives

Marine cyanobacteria are arguably one of the most important groups of creatures ever to appear on our planet. Their morphological differentiation and organized structures have led them to occupy important ecological niches since their first appearance in ancient ecosystems. Cyanobacteria have appeared in the fossil record since *Eoentophysalis belcherensis* Hofmann, early in the Proterozoic Eon, and their appearances throughout multiple eras have been keenly documented by scientists, assisting in understanding the complexity and diversity of evolutionary history. Today, marine cyanobacteria represent a significant source of structurally varied and powerful pharmaceutical agents. Herein, we screened studies on 83 bioactive compounds conducted between 2017 and 2022. In this article, the collected compounds are reported as possessing potent activities, such as cytotoxic activity, anti-trypanosomal activity, antibacterial activity, serine protease inhibition, and anticancer activity. These compounds belong to various classes, such as cyclic peptides, linear lipopeptides, depsipeptides, and cyclodepsipeptides, which have been isolated from different sources, such as *Schizothrix*, *Moorea*, *Okeania*, and *Lyngbya* genera. From the statistical findings conducted on the screened bioactive compounds, we conclude that about 43% of these have cytotoxic activity with an IC_50_ > 10 µM, and about 8% have anti-trypanosomal effects with IC_50_ values > 10 µM, compared with the positive control. We also found that most of the bioactive compounds isolated are from peptides, particularly depsipeptides. 

Clinically, we discovered that marine cyanobacteria have been the subject of various clinical studies at differing stages, some of which were inapplicable, and others were still being studied. Some of these studies, such as those on Spirulina platensis, Aphanizomenon Flos-aquae, Apomivir^®^, and Phycocare^®^ have been experimented with to treat various disorders, such as hepatitis B virus, seasonal influenza viruses, metastatic gastric cancer, and congestive heart failure. Nitrogen fixation is one of the main biological properties of marine cyanobacteria. *Trichodesmium* is the dominant nitrogen fixer on the planet and amounts to approximately 50% of global natural N_2_ fixation. It has differentiated cells that can fix nitrogen by reducing N_2_ to ammonia (NH_3_) or ammonium (NH_4_^+^). Other groups of marine cyanobacteria, including unicellular cyanobacterium and filamentous heterocyst-forming symbionts, are unable to fix nitrogen on their own. Furthermore, their fixation processes remain unclear, as does the role that marine cyanobacteria play in fixing atmospheric carbon. The effects of climate change on marine cyanobacteria require further investigation. Climate change plays a role in cyanobacteria, increasing their production of CO_2_ and enhancing nitrogen fixation. Therefore, cyanobacteria are considered one of the saviors for the planet from climate changes.

In this regard, and to expand on the exploration of cyanobacteria, a database of the newly identified marine cyanobacterial organisms, and their inclusion as a part of modern taxonomy, would help the understanding of their evolutionary pathway. The recognition of the most potent bioactive compounds with cytotoxic effects and their potential to be tested in clinical studies will require more in-depth investigation and preclinical trials, including in-vivo and in situ experimental designs. Ultimately, more studies will be needed to explain the mechanisms underlying nitrogen fixation. 

## Figures and Tables

**Table 8:** Survey of marine cyanobacteria passed into different phases of clinical trials between 2017 and 2022.

Intervention or Treatment/Form	Condition or/Disease	Type of the Study/Clinical Trials Phase/No. of Participants	Dose	Administration Route/Effects	References ClinicalTrials.GovIdentifier
Apomivir^®^/Capsule	Influenza	Interventional/Phase 2/196 participants	Apomivir^®^: (120 mg b.i.d.) 1 capsule twice daily Placebo: 1 capsule twice daily	Orally after meal/Apomivir^®^ has been proven to have excellent broad-spectrum antiviral ability, especially for seasonal influenza viruses (Influenza virus A and B)	(https://clinicaltrials.gov (accessed on 15 April 2023), NCT01677689)
Dietary Supplement: Water-soluble Extract of the cyanobacteria *Aphanizomenon* Flos-aquae (AFA)/Capsules	Congestive heart failure, chronic	Observational/Not reported/15 participants	2 capsules 3 time a day	Oral/The improvement in patient’s quality of life after 12 months of trial admission	(https://clinicaltrials.gov accessed on 15 April 2023), NCT04515537)
Phycocyanin (Phycocare^®^) Other: Placebo/Not reported	Metastatic gastric cancer	Interventional/Not Applicable/110 participants	Phycocare: Every day for 9 months (except days of chemotherapy: no Phycocare) Placebo: Every day for 9 months (except days of chemotherapy = no Placebo)	Not reported/phycocyanin may give protection against oxaliplatin-induced neuropathy	(https://clinicaltrials.gov accessed on 15 April 2023), NCT05025826)
Dietary Supplement: Immulina TM Dietary Supplement: Placebo/Capsules	Post-acute COVID-19 syndrome	Interventional/Not Applicable/120 participants	Immulina TM: (200 mg per capsule) 2–200 mg capsules given by mouth in the morning and 2–200 mg capsules given by mouth in the evening for 8 weeks durationPlacebo: As the same	Oral/Not reported	(https://clinicaltrials.gov accessed on 15 April 2023), NCT05524532)

## Data Availability

Not applicable.

## Supplementary Materials

The following supporting information can be downloaded at: https://www.mdpi.com/article/10.3390/md21080439/s1, Table S1: Spectral analysis of isolated bioactive compounds with cytotoxicity targets between (2017 to 2022); Table S2: Spectral analysis of isolated bioactive compounds with antiparasitic targets between (2017 to 2022); Table S3: Spectral analysis of isolated bioactive compounds with serine protease inhibition targets between (2017 to 2022); Table S4: Spectral analysis of isolated bioactive compounds with antiproliferation and anticancer targets between (2017 to 2022); Table S5: Spectral analysis of isolated bioactive compounds with anti-quorum-sensing activity between (2017 to 2022); Table S6: Spectral analysis of isolated bioactive compounds with antibacterial activity between (2017 to 2022); Table S7: Spectral analysis of isolated bioactive compounds with different activity between (2017 to 2022).
