# Supplementary material for: Review of Marine Cyanobacteria and the Aspects Related to Their Roles: Chemical, Biological Properties, Nitrogen Fixation and Climate Change
Source: Mar Drugs. 2023 Aug 3;21(8):439. doi: 10.3390/md21080439 (PMC10456358; doi:10.3390/md21080439)
Supplement: Supplementary file 1 [file marinedrugs-21-00439-s001.zip › marinedrugs-2455855-supplementary.pdf]

Review of Marine Cyanobacteria and the Aspects Related to Their Roles: Chemical, Biological Properties, Nitro-gen Fixation and Climate Change

Hesham R. El-Seedi [1,2,3,4,*], Mohamed F. El-Mallah [4], Nermeen Yosri [5,6], Muaaz Alajlani [7], Chao Zhao [8], Muhammad A. Mehmood [9], Ming Du [10], Hammad Ullah [11], Maria Daglia [1,11], Zhiming Guo [6], Shaden A. M. Khalifa [1,12,*] and Qiyang Shou [13,*]

[1] International Research Center for Food Nutrition and Safety, Jiangsu University, Zhenjiang 212013, China
[2] International Joint Research Laboratory of Intelligent Agriculture and Agri-Products Processing, Jiangsu University, Jiangsu Education Department, Nanjing 210024, China
[3] Department of Chemistry, Faculty of Science, Islamic University of Madinah, Madinah 42351, Saudi Arabia
[4] Department of Chemistry, Faculty of Science, Menoufia University, Shebin El-Kom 32512, Egypt; elmallahmohammed9994@yahoo.com
[5] Chemistry Department of Medicinal and Aromatic Plants, Research Institute of Medicinal and Aromatic Plants (RIMAP), Beni-Suef University, Beni-Suef 62514, Egypt; nermeen.yosri@rimp.bsu.edu.eg
[6] China Light Industry Key Laboratory of Food Intelligent Detection & Processing, School of Food and Biological Engineering, Jiangsu University, Zhenjiang 212013, China; guozhiming@ujs.edu.cn
[7] Faculty of Pharmacy, Al-Sham Private University, Damascus 0100, Syria; muaaz.alajlani.foph@aspu.edu.sy
[8] College of Marine Sciences, Fujian Agriculture and Forestry University, Fuzhou 350002, China; zhchao@live.cn
[9] Bioenergy Research Center, Department of Bioinformatics and Biotechnology, Government College University Faisalabad, Faisalabad 38000, Pakistan; draamer@gcuf.edu.pk
[10] School of Food Science and Technology, National Engineering Research Center of Seafood, Dalian Polytechnic University, Dalian 116034, China; duming@dlpu.edu.cn
[11] Department of Pharmacy, University of Napoli Federico II, Via D. Montesano 49, 80131 Naples, Italy
[12] Psychiatry and Psychology Department, Capio Saint Göran's Hospital, Sankt Göransplan 1, 112 19 Stockholm, Sweden
[13] Second Clinical Medical College, Jinhua Academy, Zhejiang Chinese Medical University, Hangzhou 310053, China
* Correspondence: hesham.el-seedi@fkog.uu.se or hesham@kth.se (H.R.E.-S.); shaden.khalifa@regionstockholm.se (S.A.M.K.); shouqiyang@zcmu.edu.cn (Q.S.)

Table S1. Spectral analysis of isolated bioactive compounds with cytotoxicity targets between (2017 to 2022).

Compound name/ Class	Spectral analysis	Reference
Samoamide A, cyclic octapeptide (**1**)	HRESIMS, IR, GNPS, 1D and 2D NMR	[1]
Odobromoamide, cyclo-depsipeptide (**2**)	ESIMS, HRESIMS, 1D and 2D NMR	[2]
Samholide A, swinholides (**3**)	FT-IR, UV, HRESIMS, ^{1}H, ^{13}C NMR and GNPS	[3]
Samholide B, swinholides (**4**)	FT-IR, UV, HRESIMS, ^{1}H, ^{13}C NMR and GNPS	[3]
Samholide C, swinholides (**5**)	FT-IR, UV, HRESIMS, ^{1}H, ^{13}C NMR and GNPS	[3]
Samholide D, swinholides (**6**)	FT-IR, UV, HRESIMS, ^{1}H, ^{13}C NMR and GNPS	[3]
Samholide E, swinholides (**7**)	FT-IR, UV, HRESIMS, ^{1}H, ^{13}C NMR and GNPS	[3]
Samholide F, swinholides (**8**)	FT-IR, UV, HRESIMS, ^{1}H, ^{13}C NMR and GNPS	[3]
Samholide G, swinholides (**9**)	FT-IR, UV, HRESIMS, ^{1}H, ^{13}C NMR and GNPS	[3]
Samholide H, swinholides (**10**)	FT-IR, UV, HRESIMS, ^{1}H, ^{13}C NMR and GNPS	[3]
Samholide I, swinholides (**11**)	FT-IR, UV, HRESIMS, ^{1}H, ^{13}C NMR and GNPS	[3]
Kakeromamide A, cyclic pentapeptide (**12**)	HRFABMS, IR, ^{1}H NMR, HMBC, HMQC and LC-MS	[4]
Dragocin A, unique hybrid structural class (**13**)	HRESITOFMS, IR, 1D and 2D NMR	[5]
Dragocin B, unique hybrid structural class (**14**)	HRESITOFMS, IR, 1D and 2D NMR	[5]
Dragocin C, unique hybrid structural class (**15**)	HRESITOFMS, IR, 1D and 2D NMR	[5]
Dragocin D, unique hybrid structural class (**16**)	HRESITOFMS, IR, 1D and 2D NMR	[5]
Microcolin E, linear lipopeptide (**17**)	LC-UV-MS/MS, ^{1}H and ^{13}C NMR	[6]
Microcolin F, linear lipopeptides (**18**)	LC-UV-MS/MS, ^{1}H and ^{13}C NMR	[6]
Microcolin G, linear lipopeptides (**19**)	LC-UV-MS/MS, ^{1}H and ^{13}C NMR	[6]
Microcolin H, linear lipopeptides (**20**)	LC-UV-MS/MS, ^{1}H and ^{13}C NMR	[6]
Microcolin I, linear lipopeptides (**21**)	LC-UV-MS/MS, ^{1}H and ^{13}C NMR	[6]
Microcolin J, linear lipopeptides (**22**)	LC-UV-MS/MS, ^{1}H and ^{13}C NMR	[6]
Microcolin K, linear lipopeptides (**23**)	LC-UV-MS/MS, ^{1}H and ^{13}C NMR	[6]
Microcolin L, linear lipopeptides (**24**)	LC-UV-MS/MS, ^{1}H and ^{13}C NMR	[6]
Microcolin M, linear lipopeptides (**25**)	LC-UV-MS/MS, ^{1}H and ^{13}C NMR	[6]
Oscillatoxin I, aplysiatoxin (**26**)	1D, 2D NMR, UV and HR-ESI-MS	[7]

Table S2. Spectral analysis of isolated bioactive compounds with antiparasitic targets between (2017 to 2022).

Compound name/ Class	Spectral analysis	Reference
Dudawalamide A, cyclic depsipeptide (**27**)	LC-HRMS, HRESIMS, ^{1}H, ^{13}C, 2D NMR and DSQ/TRACE-GC-Ultra GCMS	[8]
Dudawalamide B, cyclic depsipeptide (**28**)	LC-HRMS, HRESIMS, ^{1}H, ^{13}C, 2D NMR and DSQ/TRACE-GC-Ultra GCMS	[8]
Dudawalamide C, cyclic depsipeptide (**29**)	LC-HRMS, HRESIMS, ^{1}H, ^{13}C, 2D NMR and DSQ/TRACE-GC-Ultra GCMS	[8]
Dudawalamide D, cyclic depsipeptide (**30**)	LC-HRMS, HRESIMS, ^{1}H, ^{13}C, 2D NMR and DSQ/TRACE-GC-Ultra GCMS	[8]
Hoshinolactam, contain cyclopropane ring and a γ-lactam ring system (**31**)	HRESIMS, ^{1}H, ^{13}C, COSY, HMQC, HMBC, and NOESY NMR	[9]
Mabuniamide, lipopeptide (**32**)	^{1}H NMR, ^{13}C NMR, UV and HRESIMS	[10]
Kakeromamide B, cyclic peptide (**33**)	HRMS, qNMR, ^{1}H, ^{13}C, COSY, HSQC, and HMBC NMR	[11]

Iheyamide A, linear peptides (**34**)	HRESIMS, 1D, 2D NMR, UV and IR	[12]
Iheyamide B, linear peptides (**35**)	HRESIMS, 1D, 2D NMR, UV and IR	[12]
Iheyamide C, linear peptides (**36**)	HRESIMS, 1D, 2D NMR, UV and IR	[12]
Bromoiesol sulfate A, polyhalogenated aryl sulfates (**37**)	IR, ^{1}H NMR, ^{13}C NMR, TLC, HRESIMS, X rays, HMBC, HMQC and HRAPCIMS	[13]
Bromoiesol sulfate B, polyhalogenated aryl sulfates (**38**)	IR, ^{1}H NMR, ^{13}C NMR, TLC, HRESIMS, X rays, HMBC, HMQC and HRAPCIMS	[13]
Bromoiesol A, polyhalogenated aryl (**39**)	IR, ^{1}H NMR, ^{13}C NMR, TLC, HRESIMS, X rays, HMBC, HMQC and HRAPCIMS	[13]
Bromoiesol B, polyhalogenated aryl (**40**)	IR, ^{1}H NMR, ^{13}C NMR, TLC, HRESIMS, X rays, HMBC, HMQC and HRAPCIMS	[13]
Motobamide, cyclic peptide (**41**)	Optical rotations, UV, IR, ^{1}H NMR, ^{13}C NMR, HMQC, NOESY, HMBC and HRESIMS	[14]

Table S3. Spectral analysis of isolated bioactive compounds with serine protease inhibition targets between (2017 to 2022).

Compound name/ Compound description	Spectral analysis	Reference
Grassystatin D, peptides (**42**)	^{1}H, ^{13}C, 2D NMR, LCMS, HRESIMS and LC-TOF	[15]
Grassystatin E, peptides (**43**)	^{1}H, ^{13}C, 2D NMR, LCMS, HRESIMS and LC-TOF	[15]
Grassystatin F, peptides (**44**)	^{1}H, ^{13}C, 2D NMR, LCMS, HRESIMS and LC-TOF	[15]
Jizanpeptin A, depsipeptides (**45**)	HRTOFMS, LC-MS/MS, ^{1}H and ^{13}C NMR	[16]
Jizanpeptin B, depsipeptides (**46**)	HRTOFMS, LC-MS/MS, ^{1}H and ^{13}C NMR	[16]
Jizanpeptin C, depsipeptides (**47**)	HRTOFMS, LC-MS/MS, ^{1}H and ^{13}C NMR	[16]
Jizanpeptin D, depsipeptides (**48**)	HRTOFMS, LC-MS/MS, ^{1}H and ^{13}C NMR	[16]
Jizanpeptin E, depsipeptides (**49**)	HRTOFMS, LC-MS/MS, ^{1}H and ^{13}C NMR	[16]
Tutuilamide A, cyclic peptides (**50**)	1D, 2D NMR and HR-ESI-MS	[17]
Tutuilamides B, cyclic peptides (**51**)	1D, 2D NMR and HR-ESI-MS	[17]
Tutuilamide C, cyclic peptides (**52**)	1D, 2D NMR and HR-ESI-MS	[17]

Table S4. Spectral analysis of isolated bioactive compounds with antiproliferation and anticancer targets between (2017 to 2022).

Compound name/ Compound description	Spectral analysis	Reference
Benderamide A, cyclic depsipeptide (**53**)	HRESIMS, 1D and 2D NMR	[18]
Lagunamide D, macrocyclic depsipeptide (**54**)	1D, 2D NMR and HRMS	[19]
Portobelamide A, cyclic-depsipeptides (**55**)	Optical rotations, UV, FTIR, LC-MS/MS, 1D, 2D NMR and HRESIMS	[20]
Portobelamide B, cyclic-depsipeptides(**56**)	Optical rotations, UV, FTIR, LC-MS/MS, 1D, 2D NMR and HRESIMS	[20]
Caciqueamide, long chain lipopeptide (**57**)	Optical rotations, UV, FTIR, LC-MS/MS, 1D, 2D NMR and HRESIMS	[20]
Dysidazirine carboxylic acid, carboxylic acid (**58**)	Optical rotations, UV, FTIR, ^{1}H NMR, ^{13}C NMR, HSQC, COSY, HMBC and HRESI/TOFMS	[21]

Table S5. Spectral analysis of isolated bioactive compounds with anti-quorum-sensing activity between (2017 to 2022).

Compound name/ Compound description	Spectral analysis	Reference

Trikoramide B, Decapeptide (**59**)	Optical rotations, UV, FTIR, ^{1}H NMR, ^{13}C NMR, NOESY NMR and HRMS/MS	[22]
Trikoramide C, Decapeptide (**60**)	Optical rotations, UV, FTIR, ^{1}H NMR, ^{13}C NMR, NOESY NMR and HRMS/MS	[22]
Trikoramide D, Decapeptide (**61**)	Optical rotations, UV, FTIR, ^{1}H NMR, ^{13}C NMR, NOESY NMR and HRMS/MS	[22]
Trikoveramide A, cyclic depsipeptides (**62**)	Optical rotations, UV, FTIR, ^{1}H NMR, ^{13}C NMR, HMBC and HR-ESI-Orbitrap MS	[23]
Trikoveramide B, cyclic depsipeptides (**63**)	Optical rotations, UV, FTIR, ^{1}H NMR, ^{13}C NMR, HMBC and HR-ESI-Orbitrap MS	[23]
Trikoveramide C, cyclic depsipeptides (**64**)	Optical rotations, UV, FTIR, ^{1}H NMR, ^{13}C NMR, HMBC and HR-ESI-Orbitrap MS	[23]

Table S6. Spectral analysis of isolated bioactive compounds with antibacterial activity between (2017 to 2022).

**Compound name/ Compound description**	**Spectral analysis**	**Reference**
2-hydroxyethyl-11-hydroxyhexadec-9-enoate, Not Reported (**65**)	HR-ESIMS, FT-IR, ^{1}H, ^{13}C and 2D NMR	[24]
Tiahuramides A, cyclic depsipeptides (**66**)	HRMS, IR, FTIR, UV, 1D and 2D NMR	[25]
Tiahuramide B, cyclic depsipeptides (**67**)	HRMS, IR, FTIR, UV, 1D and 2D NMR	[25]
Tiahuramide C, cyclic depsipeptides (**68**)	HRMS, IR, FTIR, UV, 1D and 2D NMR	[25]

Table S7. Spectral analysis of isolated bioactive compounds with different activity between (2017 to 2022).

**Compound name/ Compound description**	**Spectral analysis**	**Reference**
Biseokeaniamide A, linear lipopeptides (**69**)	IR, ^{1}H, ^{13}C NMR, HMQC, COSY, NOESY, HMBC and HRESIMS	[26]
Biseokeaniamide B, linear lipopeptides (**70**)	IR, ^{1}H, ^{13}C NMR, HMQC, COSY, NOESY, HMBC and HRESIMS	[26]
Biseokeaniamide C, linear lipopeptides (**71**)	IR, ^{1}H, ^{13}C NMR, HMQC, COSY, NOESY, HMBC and HRESIMS	[26]
Serinolamide C, fatty acid amides (**72**)	ESITOFMS, ^{1}H, ^{13}C, 2D NMR and LC-MS	[27]
Serinolamide D, fatty acid amides (**73**)	ESITOFMS, ^{1}H, ^{13}C, 2D NMR and LC-MS	[27]
Lyngbyabellin O, Not reported (**74**)	ESITOFMS, ^{1}H, ^{13}C, 2D NMR and LC-MS	[27]
Lyngbyabellin P, Not reported (**75**)	ESITOFMS, ^{1}H, ^{13}C, 2D NMR and LC-MS	[27]
Columbamide D, chlorinated fatty acid amides (**76**)	ESI-FTMS, 1D and 2D NMR	[28]
Columbamide E, chlorinated fatty acid amides (**77**)	ESI-FTMS, 1D and 2D NMR	[28]
6,8-di-*O*-acetylmal-yngamide 2, Malyngamide series (**78**)	HRESIMS, 1D and 2D NMR	[29]
6-*O*-acetylmal-yngamide 2, Malyngamide series (**79**)	HRESIMS, 1D and 2D NMR	[29]
N-demethyl-isomal-yngamide I, Malyngamide series (**80**)	HRESIMS, 1D and 2D NMR	[29]
Neo-debromoaplysi-atoxin A, polyketides (**81**)	HR-ESI-MS, UV, X-ray, 1D and 2D NMR	[30]
Neo-debromoaplysi-atoxin B, polyketides (**82**)	HR-ESI-MS, X-ray, 1D and 2D NMR	[30]

Croissamide, cyclic peptide (**83**)	HRESIMS, ^{1}H NMR, ^{13}C NMR, COSY, TOCSY, HMQC, HMBC and NOESY	[31]

References

1. Naman, C.B.; Rattan, R.; Nikoulina, S.E.; Lee, J.; Miller, B.W.; Moss, N.A.; Armstrong, L.; Boudreau, P.D.; Debonsi, H.M.; Valeriote, F.A.; et al. Integrating Molecular Networking and Biological Assays To Target the Isolation of a Cytotoxic Cyclic Octapeptide, Samoamide A, from an American Samoan Marine Cyanobacterium. *J. Nat. Prod.* **2017**, *80*, 625–633, doi:10.1021/acs.jnatprod.6b00907.

2. Sueyoshi, K.; Kudo, T.; Yamano, A.; Sumimoto, S.; Iwasaki, A.; Suenaga, K.; Teruya, T. Odobromoamide, a terminal alkynyl bromide-containing cyclodepsipeptide from the marine cyanobacterium Okeania sp. *Bull. Chem. Soc. Jpn.* **2017**, *90*, 436–440.

3. Tao, Y.; Li, P.; Zhang, D.; Glukhov, E.; Gerwick, L.; Zhang, C.; Murray, T.F.; Gerwick, W.H. Samholides, Swinholide-Related Metabolites from a Marine Cyanobacterium cf. Phormidium sp. *J. Org. Chem.* **2018**, *83*, 3034–3046, doi:10.1021/acs.joc.8b00028.

4. Nakamura, F.; Maejima, H.; Kawamura, M.; Arai, D.; Okino, T.; Zhao, M.; Ye, T.; Lee, J.; Chang, Y.-T.; Fusetani, N.; et al. Kakeromamide A, a new cyclic pentapeptide inducing astrocyte differentiation isolated from the marine cyanobacterium Moorea bouillonii. *Bioorg. Med. Chem. Lett.* **2018**, *28*, 2206–2209, doi:https://doi.org/10.1016/j.bmcl.2018.04.067.

5. Choi, H.; Engene, N.; Byrum, T.; Hwang, S.; Oh, D.-C.; Gerwick, W.H. Dragocins A−D, Structurally Intriguing Cytotoxic Metabolites from a Panamanian Marine Cyanobacterium. *Org. Lett.* **2019**, *21*, 266–270, doi:10.1021/acs.orglett.8b03712.

6. Yu, H.-B.; Glukhov, E.; Li, Y.; Iwasaki, A.; Gerwick, L.; Dorrestein, P.C.; Jiao, B.-H.; Gerwick, W.H. Cytotoxic Microcolin Lipopeptides from the Marine Cyanobacterium Moorea producens. *J. Nat. Prod.* **2019**, *82*, 2608–2619, doi:10.1021/acs.jnatprod.9b00549.

7. Nagai, H.; Sato, S.; Iida, K.; Hayashi, K.; Kawaguchi, M.; Uchida, H.; Satake, M. Oscillatoxin I: A New Aplysiatoxin Derivative, from a Marine Cyanobacterium. *Toxins (Basel).* **2019**, *11*, 366, doi:10.3390/toxins11060366.

8. Almaliti, J.; Malloy, K.L.; Glukhov, E.; Spadafora, C.; Gutiérrez, M.; Gerwick, W.H. Dudawalamides A–D, Antiparasitic Cyclic Depsipeptides from the Marine Cyanobacterium Moorea producens. *J. Nat. Prod.* **2017**, *80*, 1827–1836, doi:10.1021/acs.jnatprod.7b00034.

9. Ogawa, H.; Iwasaki, A.; Sumimoto, S.; Iwatsuki, M.; Ishiyama, A.; Hokari, R.; Otoguro, K.; Ōmura, S.; Suenaga, K. Isolation and total synthesis of hoshinolactam, an antitrypanosomal lactam from a marine cyanobacterium. *Org. Lett.* **2017**, *19*, 890–893.

10. Ozaki, K.; Iwasaki, A.; Sezawa, D.; Fujimura, H.; Nozaki, T.; Saito-Nakano, Y.; Suenaga, K.; Teruya, T. Isolation and Total Synthesis of Mabuniamide, a Lipopeptide from an Okeania sp. Marine Cyanobacterium. *J. Nat. Prod.* **2019**, *82*, 2907–2915, doi:10.1021/acs.jnatprod.9b00749.

11. Sweeney-Jones, A.M.; Gagaring, K.; Antonova-Koch, J.; Zhou, H.; Mojib, N.; Soapi, K.; Skolnick, J.; McNamara, C.W.; Kubanek, J. Antimalarial Peptide and Polyketide Natural Products from the Fijian Marine Cyanobacterium Moorea producens. *Mar. Drugs* **2020**, *18*, 167,

doi:10.3390/md18030167.

12. Kurisawa, N.; Iwasaki, A.; Jeelani, G.; Nozaki, T.; Suenaga, K. Iheyamides A–C, Antitrypanosomal Linear Peptides Isolated from a Marine Dapis sp. Cyanobacterium. *J. Nat. Prod.* **2020**, *83*, 1684–1690, doi:10.1021/acs.jnatprod.0c00250.

13. Ebihara, A.; Iwasaki, A.; Miura, Y.; Jeelani, G.; Nozaki, T.; Suenaga, K. Isolation and Total Synthesis of Bromoiesol sulfates, Antitrypanosomal arylethers from a Salileptolyngbya sp. Marine Cyanobacterium. *J. Org. Chem.* **2021**, *86*, 11763–11770, doi:10.1021/acs.joc.1c01214.

14. Takahashi, H.; Iwasaki, A.; Kurisawa, N.; Suzuki, R.; Jeelani, G.; Matsubara, T.; Sato, T.; Nozaki, T.; Suenaga, K. Motobamide, an Antitrypanosomal Cyclic Peptide from a Leptolyngbya sp. Marine Cyanobacterium. *J. Nat. Prod.* **2021**, *84*, 1649–1655, doi:10.1021/acs.jnatprod.1c00234.

15. Al-Awadhi, F.H.; Law, B.K.; Paul, V.J.; Luesch, H. Grassystatins D–F, Potent Aspartic Protease Inhibitors from Marine Cyanobacteria as Potential Antimetastatic Agents Targeting Invasive Breast Cancer. *J. Nat. Prod.* **2017**, *80*, 2969–2986, doi:10.1021/acs.jnatprod.7b00551.

16. Gallegos, D.A.; Saurí, J.; Cohen, R.D.; Wan, X.; Videau, P.; Vallota-Eastman, A.O.; Shaala, L.A.; Youssef, D.T.A.; Williamson, R.T.; Martin, G.E.; et al. Jizanpeptins, Cyanobacterial Protease Inhibitors from a Symploca sp. Cyanobacterium Collected in the Red Sea. *J. Nat. Prod.* **2018**, *81*, 1417–1425, doi:10.1021/acs.jnatprod.8b00117.

17. Keller, L.; Canuto, K.M.; Liu, C.; Suzuki, B.M.; Almaliti, J.; Sikandar, A.; Naman, C.B.; Glukhov, E.; Luo, D.; Duggan, B.M.; et al. Tutuilamides A–C: Vinyl-Chloride-Containing Cyclodepsipeptides from Marine Cyanobacteria with Potent Elastase Inhibitory Properties. *ACS Chem. Biol.* **2020**, *15*, 751–757, doi:10.1021/acschembio.9b00992.

18. Ding, C.Y.; Ong, J.F.; Goh, H.C.; Coffill, C.R.; Tan, L.T. Benderamide A, a Cyclic Depsipeptide from a Singapore Collection of Marine Cyanobacterium cf. Lyngbya sp. *Mar. Drugs* 2018, *16*.

19. Luo, D.; Putra, M.; Ye, T.; Paul, V.; Luesch, H. Isolation, Structure Elucidation and Biological Evaluation of Lagunamide D: A New Cytotoxic Macrocyclic Depsipeptide from Marine Cyanobacteria. *Mar. Drugs* **2019**, *17*, 83, doi:10.3390/md17020083.

20. Demirkiran, O.; Almaliti, J.; Leão, T.; Navarro, G.; Byrum, T.; Valeriote, F.A.; Gerwick, L.; Gerwick, W.H. Portobelamides A and B and Caciqueamide, Cytotoxic Peptidic Natural Products from a Caldora sp. Marine Cyanobacterium. *J. Nat. Prod.* **2021**, *84*, 2081–2093, doi:10.1021/acs.jnatprod.0c01383.

21. Gunasekera, S.P.; Kokkaliari, S.; Ratnayake, R.; Sauvage, T.; dos Santos, L.A.H.; Luesch, H.; Paul, V.J. Anti-Inflammatory Dysidazirine Carboxylic Acid from the Marine Cyanobacterium Caldora sp. Collected from the Reefs of Fort Lauderdale, Florida. *Mol.* 2022, *27*.

22. Phyo, M.Y.; Goh, T.M.; Goh, J.X.; Tan, L.T. Trikoramides B–D, Bioactive Cyanobactins from the Marine Cyanobacterium Symploca hydnoides. *Mar. Drugs* 2021, *19*.

23. Phyo, M.Y.; Katermeran, N.P.; Goh, J.X.; Tan, L.T. Trikoveramides A-C, cyclic depsipeptides from the marine cyanobacterium Symploca hydnoides. *Phytochemistry* **2021**, *190*, 112879, doi:https://doi.org/10.1016/j.phytochem.2021.112879.

24. Maneechote, N.; Yingyongnarongkul, B.; Suksamran, A.; Lumyong, S. Inhibition of Vibrio spp. by 2-Hydroxyethyl-11-hydroxyhexadec-9-enoate of Marine Cyanobacterium Leptolyngbya sp. LT19.

Aquac. Res. **2017**, *48*, 2088–2095, doi:10.1111/are.13043.

25. Levert, A.; Alvariño, R.; Bornancin, L.; Abou Mansour, E.; Burja, A.M.; Genevière, A.-M.; Bonnard, I.; Alonso, E.; Botana, L.; Banaigs, B. Structures and Activities of Tiahuramides A–C, Cyclic Depsipeptides from a Tahitian Collection of the Marine Cyanobacterium Lyngbya majuscula. *J. Nat. Prod.* **2018**, *81*, 1301–1310, doi:10.1021/acs.jnatprod.7b00751.

26. Iwasaki, A.; Tadenuma, T.; Sumimoto, S.; Ohshiro, T.; Ozaki, K.; Kobayashi, K.; Teruya, T.; Tomoda, H.; Suenaga, K. Biseokeaniamides A, B, and C, sterol O-acyltransferase inhibitors from an Okeania sp. marine cyanobacterium. *J. Nat. Prod.* **2017**, *80*, 1161–1166.

27. Petitbois, J.G.; Casalme, L.O.; Lopez, J.A. V; Alarif, W.M.; Abdel-Lateff, A.; Al-Lihaibi, S.S.; Yoshimura, E.; Nogata, Y.; Umezawa, T.; Matsuda, F.; et al. Serinolamides and Lyngbyabellins from an Okeania sp. Cyanobacterium Collected from the Red Sea. *J. Nat. Prod.* **2017**, *80*, 2708–2715, doi:10.1021/acs.jnatprod.7b00449.

28. Lopez, J.A. V; Petitbois, J.G.; Vairappan, C.S.; Umezawa, T.; Matsuda, F.; Okino, T. Columbamides D and E: Chlorinated Fatty Acid Amides from the Marine Cyanobacterium Moorea bouillonii Collected in Malaysia. *Org. Lett.* **2017**, *19*, 4231–4234, doi:10.1021/acs.orglett.7b01869.

29. Sueyoshi, K.; Yamano, A.; Ozaki, K.; Sumimoto, S.; Iwasaki, A.; Suenaga, K.; Teruya, T. Three new malyngamides from the marine cyanobacterium Moorea producens. *Mar. Drugs* **2017**, *15*, 367.

30. Han, B.-N.; Liang, T.-T.; Keen, L.J.; Fan, T.-T.; Zhang, X.-D.; Xu, L.; Zhao, Q.; Wang, S.-P.; Lin, H.-W. Two Marine Cyanobacterial Aplysiatoxin Polyketides, Neo-debromoaplysiatoxin A and B, with K+ Channel Inhibition Activity. *Org. Lett.* **2018**, *20*, 578–581, doi:10.1021/acs.orglett.7b03672.

31. Iwasaki, K.; Iwasaki, A.; Sumimoto, S.; Sano, T.; Hitomi, Y.; Ohno, O.; Suenaga, K. Croissamide, a proline-rich cyclic peptide with an N-prenylated tryptophan from a marine cyanobacterium Symploca sp. *Tetrahedron Lett.* **2018**, *59*, 3806–3809.
